# Thioredoxin reductase 1 suppresses adipocyte differentiation and insulin responsiveness

**DOI:** 10.1038/srep28080

**Published:** 2016-06-27

**Authors:** Xiaoxiao Peng, Alfredo Giménez-Cassina, Paul Petrus, Marcus Conrad, Mikael Rydén, Elias S. J. Arnér

**Affiliations:** 1 Division of Biochemistry, Department of Medical Biochemistry and Biophysics, Karolinska Institutet, SE-171 77 Stockholm, Sweden; 2 Departamento de Biología Molecular, Universidad Autónoma de Madrid, Centro de Biología Molecular “Severo Ochoa” (CSIC-UAM), 28049, Madrid, Spain; 3 Clinical Research Center, and the Department of Medicine, Huddinge University Hospital, Karolinska Institutet, SE-141 86 Stockholm, Sweden; 4 Helmholtz Zentrum München, Institute of Developmental Genetics, Ingolstädter Landstrasse 1, 85764 Neuherberg, Germany

## Abstract

Recently thioredoxin reductase 1 (TrxR1), encoded by *Txnrd1*, was suggested to modulate glucose and lipid metabolism in mice. Here we discovered that TrxR1 suppresses insulin responsiveness, anabolic metabolism and adipocyte differentiation. Immortalized mouse embryonic fibroblasts (MEFs) lacking *Txnrd1 (Txnrd1*^−/−^) displayed increased metabolic flux, glycogen storage, lipogenesis and adipogenesis. This phenotype coincided with upregulated PPARγ expression, promotion of mitotic clonal expansion and downregulation of p27 and p53. Enhanced Akt activation also contributed to augmented adipogenesis and insulin sensitivity. Knockdown of *TXNRD1* transcripts accelerated adipocyte differentiation also in human primary preadipocytes. Furthermore, *TXNRD1* transcript levels in subcutaneous adipose tissue from 56 women were inversely associated with insulin sensitivity *in vivo* and lipogenesis in their isolated adipocytes. These results suggest that TrxR1 suppresses anabolic metabolism and adipogenesis by inhibition of intracellular signaling pathways downstream of insulin stimulation.

Adipose tissue plays a major role in energy metabolism, and aberrations in its development or function can lead to an array of metabolic alterations and diseases. Up to 90% of the volume of adipose tissue is comprised of adipocytes generated through adipogenesis from specific precursor cells and in adult humans, white adipose tissue is in a dynamic state where approximately 10% of the adipocyte pool is turned over annually^1^. The process of adipogenesis requires an orchestrated multistep process of sequentially concerted signaling events, converging on activation of key transcription factors required for adipocyte formation. The most critical transcription factors include members of the CCAAT-enhancer-binding protein (C/EBP) and peroxisome proliferator-activated receptor (PPAR) families^2^,^3^. Early signaling events mediated by C/EBPβ and C/EBPα contribute to initiate adipogenesis by inducing PPARγ, among others. In addition, they also contribute to maintenance of the adipose phenotype^3^. Activation of the nuclear receptor PPARγ is both necessary and sufficient for differentiation of adipocytes^4^ and pro-adipogenic or anti-adipogenic factors either induce or repress PPARγ, respectively^3^. Adipocyte differentiation is also closely linked to insulin signaling^3^. Attenuating insulin signaling, for instance by loss of insulin-receptor substrate (IRS) proteins, inhibition of phosphatidylinositol-3 kinase (PI3K) or depletion of AKT/protein kinase B (PKB), leads to suppression of adipocyte differentiation^5^,^6^,^7^.

The mammalian selenoprotein thioredoxin reductase 1 (TrxR1), encoded in mice by *Txnrd1* and in human by *TXNRD1*, plays a key role in antioxidant defense, redox regulation, cell proliferation and cell signaling events, by catalyzing reduction of the active site disulfide of thioredoxin 1 (Trx1) and several other substrates in cells^8^. Germline deletion of *Txnrd1* in mice causes early embryonic death^9^,^10^. However, hepatocyte-specific conditional deletion of *Txnrd1* is not lethal, but results in pronounced alterations of glycogen and lipid storage in the liver^11^,^12^. Although somewhat conflicting data have been published, some observations indicate that TrxR1 can influence lipid turnover. Hepatocyte-specific disruption of *Txnrd1* was found in one study to cause a metabolic switch in which hepatic lipogenesis seemed to be repressed and glycogen storage greatly increased^11^, while another study reported mild to severe hepatic accumulation of lipids^12^. In the study by Iverson and colleagues, lipid content was assessed using transmission electron microscopy (TEM) and appeared repressed in periportal hepatocytes because of high glycogen accumulation^11^. In the Carlson study, lipids were assessed using morphological assessments of hepatocyte vacuoles that were judged to resemble lipid vesicles^12^. Thereby neither of these studies validated fat accumulation by direct measurements of lipid content. Thus, while both studies suggested a role of TrxR1 in regulation of glucose and/or lipid metabolism, effects of TrxR1 on lipid metabolism clearly remain to be defined.

Here, we first examined the impact of TrxR1 on glucose and lipid metabolism using well-defined cell culture models optimally suited for studies of molecular mechanisms. Because primary MEFs can be differentiated into osteocytes, chondrocytes or adipocytes depending upon choice of hormonal stimuli^13^, we studied the propensity of *Txnrd1*^−/−^ MEFs to undergo adipogenesis and compared this to parental cells expressing the intact *Txnrd1* gene. We also determined adipocyte differentiation of primary human preadipocytes transfected with *TXNRD1-*targeting or non silencing siRNA oligonucleotides, and analyzed possible correlations of *TXNRD1* transcript levels to clinical parameters in a cohort of obese and non-obese women. Our results collectively suggest that TrxR1 exerts a hitherto unknown but potent role in regulation of insulin responsiveness and adipogenesis.

## Results

### *Txnrd1* deletion leads to altered glucose handling in immortalized mouse embryonic fibroblasts

**Figure 1:**
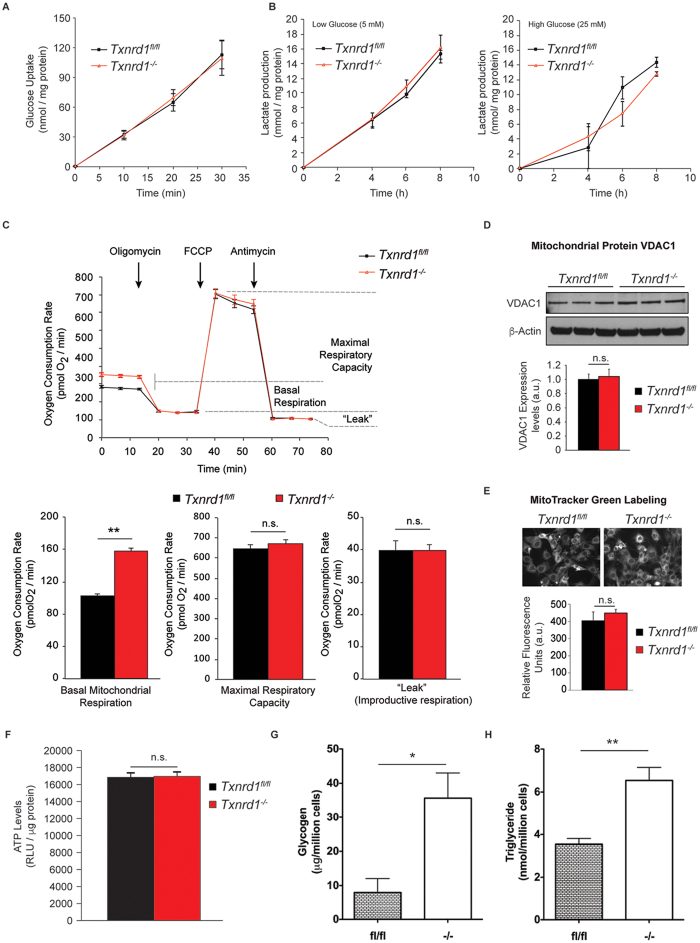
TrxR1 depletion promotes glucose utilization in biosynthetic pathways. (**A**) Glucose uptake in *Txnrd1*^*fl*/*fl*^ and *Txnrd1*^−/−^ MEFs (n = 3, ±SEM). (**B**) Glycolytic flux in MEFs, as determined by lactate release to the extracellular milieu (n = 3, ±SEM). Given the previously described sensitivity of *Txnrd1*^−/−^ MEFs to variation in extracellular glucose levels, lactate production was measured in conditions of low glucose (5 mM, left panel) and high glucose (25 mM, right panel). (**C**) Mitochondrial respiration in real time in *Txnrd1*^*fl*/*fl*^ and *Txnrd1*^−/−^ MEFs determined by extracellular flux analysis and expressed as oxygen consumption rate (OCR) (n = 6, ±SEM), depicted in the top panel. The sequential addition of the mitochondrial ATP synthase inhibitor oligomycin, the uncoupling agent Carbonyl cyanide 4-(trifluoromethoxy)phenylhydrazone (FCCP) and the respiratory complex III inhibitor antimycin allowed us to determine basal mitochondrial respiration (bottom panel, left), maximal respiratory capacity (bottom panel, middle) and “leak”, or non-ATP coupled respiration (bottom panel, right) (**D**,**E**) Mitochondrial content in *Txnrd1*^*fl*/*fl*^ and *Txnrd1*^−/−^ MEFs as determined through levels of the mitochondrial protein voltage-dependent anion channel (VDAC1; (**D**) see Supplementary Material for non-cropped immunoblots) and by relative fluorescence intensity (Relative Fluorescent Units, RFU) yielded by the non-potentiometric mitochondrial fluorescent probe MitoTracker Green (**E**). (**F**) ATP levels in *Txnrd1*^*fl*/*fl*^ and *Txnrd1*^−/−^ MEFs in steady state, expressed as Relative Luminescent Units (RLU) (n = 8, ±SEM). (**G**) Intracellular glycogen levels in *Txnrd1*^*fl*/*fl*^ and *Txnrd1*^−/−^ MEFs. (**F**) Intracellular Triglyceride levels *Txnrd1*^*fl*/*fl*^ and *Txnrd1*^−/−^ MEFs. (*p < 0.05; **p < 0.01).

The *Txnrd1*^−/−^ MEFs used herein were previously characterized in detail and confirmed to completely lack TrxR1 expression while, in contrast, the parental *Txnrd1*^*fl*/*fl*^ MEFs have a TrxR activity of ≈25 nmol/min/mg protein^14^,^15^. Both cell types are immortalized batches of MEF, where the *Txrnd1*^−/−^ genotype was obtained by *ex vivo* treatment of the *Txnrd1*^*fl*/*fl*^ cells with Tat-Cre^14^,^15^. Here we observed that glucose uptake and glycolytic flux were not affected by deletion of TrxR1 (Fig. 1A,B). However, basal mitochondrial respiration rates in the presence of glucose, but not maximal respiration or unproductive (non-ATP coupled) oxygen consumption, were significantly increased in *Txnrd1*^−/−^ MEFs (Fig. 1C), indicating higher metabolic flux without increased mitochondrial capacity or any apparent mitochondrial dysfunction. The increase in basal mitochondrial respiration was not due to an expansion in mitochondrial mass, which was comparable between *Txnrd1*^*fl*/*fl*^ and *Txnrd1*^−/−^ cells (Fig. 1D,E). Strikingly, increased mitochondrial respiration in *Txnrd1*^−/−^ cells was furthermore not correlated with higher steady-state ATP levels (Fig. 1F). This led us to hypothesize that the elevated metabolic flux in absence of TrxR1 was geared towards anabolic biosynthetic processes. Indeed, we observed that the *Txnrd1*^−/−^ cells contained significantly higher levels of glycogen (Fig. 1G) as well as triglycerides (Fig. 1H) compared to *Txnrd1*^*fl*/*fl*^ MEFs.

### The absence of TrxR1 facilitates adipocyte differentiation of immortalized mouse embryonic fibroblasts

**Figure 2:**
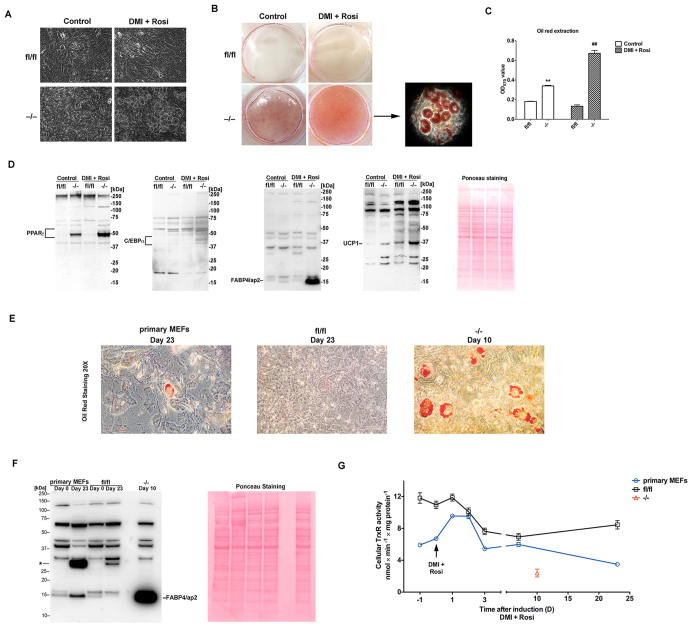
*Txnrd1* depletion increases lipogenesis and promotes adipocyte differentiation of mouse embryonic fibroblasts. (**A**) MEFs were hormonally induced to trigger adipocyte differentiation (DMI + Rosi; see Methods for details) and on day 8, the cells were imaged with 20x magnification; left column are untreated controls at day 8, right column are hormonally induced cells. (**B**) The same cells as shown in A were stained with Oil Red-O and shown are photos of the entire petri dishes; The arrow points toward an image with 20x magnification displaying the Oil Red-O-stained adipocytes formed in *Txnrd1*^−/−^ MEFs. (**C**) The Oil Red-O staining was extracted from plates as shown in B and the color intensity was measured (n = 4–8, ±SEM). Significant differences between *Txnrd1*^*fl*/*fl*^ and *Txnrd1*^−/−^ MEFs in the control samples are indicated (**P < 0.01) as well as in the hormonally treated samples (^##^P < 0.01). (**D**) The cell lysates of MEFs treated as in B were immunoblotted with antibodies against PPARγ, C/EBPα, FABP4/aP2 and UCP1. Positions of molecular weight markers (kDa) and the sizes of the analyzed proteins are as indicated. Ponceau S staining of the membrane was used as protein loading control (right). (**E**) The different MEFs were induced to adipocyte differentiation for the time indicated. Oil Red-O staining with 20x magnification was used to image the formed adipocytes. (**F**) The cell lysates of the MEFs in E were immunoblotted with antibody against FABP4/aP2, as indicated. The symbol “*” indicates an unidentified band approximately twice the size of FABP4/aP2. Ponceau S staining of the membrane was used as loading control (right). (**G**) Cellular TrxR activity in extracts of the indicated MEF cultures was measured at different time points during the adipocyte differentiation process. The arrow indicates the initiation of adipocyte differentiation using hormonal treatment.

The significantly higher content of triglycerides in *Txnrd1*^−/−^ MEFs showed that *Txnrd1* deletion facilitated fat accumulation. Remarkably, cultures of *Txnrd1*^−/−^ MEFs, unlike *Txnrd1*^*fl*/*fl*^, displayed a readily observable proportion of cells with adipocyte-like morphology containing small lipid droplets as confirmed by Oil Red-O staining (Fig. 2A,B). We therefore asked whether the enhanced ability of *Txnrd1*^−/−^ MEFs to store fat could correlate with an increased propensity for differentiation into adipocytes. To test this hypothesis, we attempted to induce adipogenic differentiation by hormonal induction using a well-established combination of pro-adipogenic factors (DMI: dexamethasone, methylisobutylxanthine and insulin, together with rosiglitazone). Indeed, *Txnrd1*^−/−^ MEFs displayed evident signs of adipocyte differentiation, which were not observed in the parental cells (Fig. 2A–C).

Because PPARγ and C/EBPα are crucial transcriptional activators in adipogenesis, we next analyzed their levels in the *Txnrd1*^−/−^ and *Txnrd1*^*fl*/*fl*^ MEFs. We found that both PPARγ and C/EBPα were readily detected in *Txnrd1*^−/−^ cells and further elevated by adipogenic induction, whereas their expression levels were much lower in the parental *Txnrd1*^*fl*/*fl*^ cells (Fig. 2D). We also examined the expression of adipocyte fatty-acid-binding protein 4 (FABP4/aP2), which is a widely used adipogenic marker^16^. Pronounced expression of FABP4/aP2 was found in hormonally induced *Txnrd1*^−/−^ MEFs, but neither in the untreated *Txnrd1*^−/−^ cells nor in the parental *Txnrd1*^*fl*/*fl*^ MEFs irrespective of hormonal treatment (Fig. 2D). Uncoupling protein 1 (UCP1) was also spontaneously expressed in the *Txnrd1*^−/−^ cells, and upregulated in both knockouts and parental cells after hormonal induction but to a higher extent in the *Txnrd1*^−/−^ MEFs (Fig. 2D). These results show that *Txnrd1*^−/−^ MEFs has a more efficient adipogenic program compared to immortalized wild type *Txnrd1*^*fl*/*fl*^ MEFs.

As we did not observe adipocyte differentiation of the parental *Txnrd1*^*fl*/*fl*^ MEFs, possibly due to the immortalization process that is well known to deprive MEFs of their potential to undergo adipogenesis^3^, we also analyzed freshly isolated primary MEFs and compared them with the *Txnrd1*^−/−^ MEFs. This demonstrated that, unlike the immortalized *Txnrd1*^*fl*/*fl*^ MEFs, primary MEFs were indeed able to differentiate into adipocytes, but only after a very long period of hormonal induction (23 days). Adipocyte differentiation of the *Txnrd1*^−/−^ MEFs was thus clearly enhanced compared also to primary MEFs, as illustrated by their stronger Oil Red-O staining and higher levels of FABP4/aP2 expression (Fig. 2E,F). Enzymatic activity assays on cell lysates harvested at different time points showed that cellular TrxR activity levels correlated inversely with the adipocyte differentiation capability of these cell types, with *Txnrd1*^*fl*/*fl*^ MEFs having 2-fold higher basal TrxR activity than primary MEFs, while the knockout cells only retained background activity, most likely representing mitochondrial thioredoxin reductase (TrxR2) activity^15^ (Fig. 2G).

### *Txnrd1* deletion accelerates mitotic clonal expansion and enhances insulin responsiveness upon induction of adipocyte differentiation

**Figure 3:**
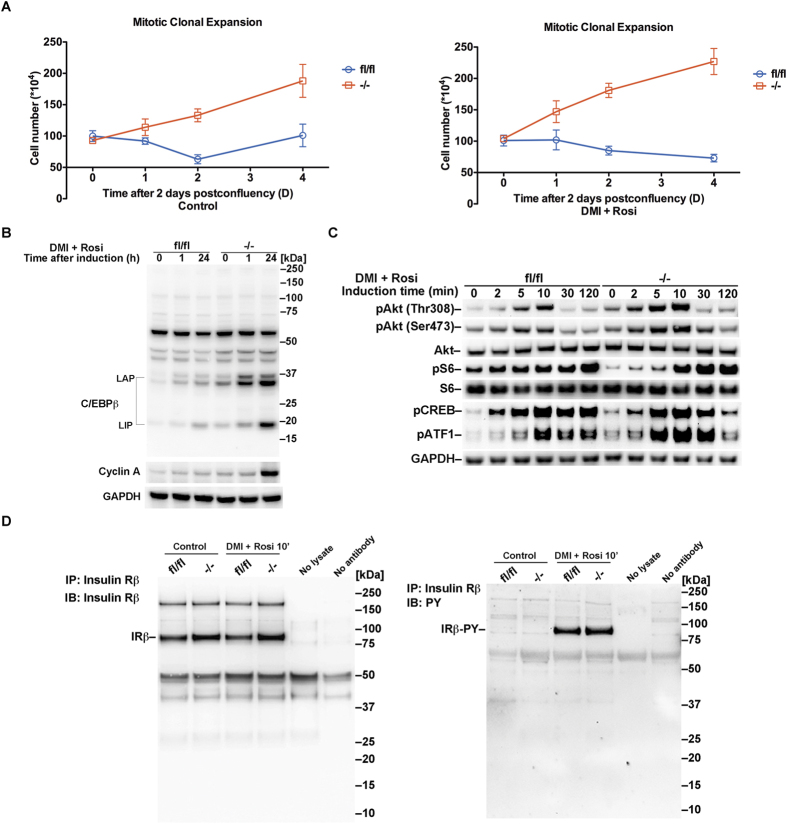
*Txnrd1* depletion causes accelerated mitotic clonal expansion and sensitizes MEFs to insulin signaling upon adipocyte differentiation induction. (**A**) *Txnrd1*^*fl*/*fl*^ and *Txnrd1*^−/−^ MEFs were either kept in culture two days after reaching confluency or hormonally induced to adipocyte differentiation as indicated and described in the text. Total cell numbers at different time points were counted and then plotted as shown (n = 4, ±SEM). Left panel are untreated controls with the right panel showing the hormonally induced cells. (**B**) The lysates of cells obtained at the different time points shown in (**A**) were immunoblotted using antibodies against C/EBPβ and cyclin A, with immunoblotting for GAPDH used as loading control. Sizes of the LAP and LIP forms of C/EBPβ are indicated. (**C**) *Txnrd1*^*fl*/*fl*^ and *Txnrd1*^−/−^ MEFs were hormonally induced for adipocyte differentiation and analyzed at different time points as indicated. The cell lysates were immunoblotted with antibodies against pAkt (Thr308), pAkt (Ser473), total Akt, pCREB, pATF1, pS6 or total S6, with immunoblotting of GAPDH used as loading control. Only the parts of the membranes corresponding to the expected sizes of the target proteins are shown. (**D**) *Txnrd1*^*fl*/*fl*^ and *Txnrd1*^−/−^ MEFs were treated for hormonal induction of adipocyte differentiation for 10 min, whereupon cell lysates were used for immunoprecipitation with an antibody against insulin receptor β subunit. The pulled down samples were subsequently immunoblotted with antibodies against the insulin receptor β subunit (left) and phosphotyrosine (PY)-containing proteins (right), as indicated. See Supplementary Material for non-cropped immunoblots.

Post-confluent and growth-arrested pre-adipocytes reenter the cell cycle to undergo two sequential rounds of mitosis following hormonal induction of differentiation, which is referred to as mitotic clonal expansion (MCE)^17^, a necessary step for adipogenesis in MEFs^18^. Here we found that the cell number in *Txnrd1*^−/−^ MEF cultures, in the absence of hormonal induction, still increased after reaching confluence to almost 2-fold after 96 h (Fig. 3A, left panel). Cell number was further increased upon hormonal induction of adipocyte differentiation (Fig. 3A, right panel). In contrast, there was no cell proliferation beyond confluency in *Txnrd1*^*fl*/*fl*^ MEFs, irrespective of hormone treatment (Fig. 3A). In agreement with these cellular phenotypes, the MCE-related transcription factor CCAAT-enhancer-binding protein β (C/EBPβ)^18^ was highly induced in *Txnrd1*^−/−^ MEFs following induction of differentiation, while this increase was much less pronounced in the parental cells (Fig. 3B). Interestingly, both the adipogenesis promoting (LAP, liver-enriched activator protein) and antiadipogenic (LIP, liver-enriched inhibitory protein) isoforms of C/EBPβ^19^ were increased in *Txnrd1*^−/−^ MEFs, but at the early time points LAP was more increased than LIP (Fig. 3B). Strong upregulation of cyclin A at 24 h after induction in the knockout cells, but not in the parental cells, was in agreement with the finding that only *Txnrd1*^−/−^ MEFs traversed the G1-S checkpoint after having reached confluency (Fig. 3B). These findings suggest that TrxR1 negatively regulates adipogenesis by facilitating contact inhibition and suppression of MCE.

Because insulin signaling has strong modulatory effects on adipogenesis we next analyzed the phosphorylation status of key intracellular phosphorylation cascade proteins linked to insulin signaling. The serine/threonine kinase Akt (also known as protein kinase B, PKB) is involved in most of the metabolic actions of insulin^20^. Here, we observed a transiently enhanced phosphorylation of Akt at residues Thr308 and Ser473 within the first hour after hormonal induction of differentiation, which was more pronounced in *Txnrd1*^−/−^ MEFs compared to the parental cells (Fig. 3C). Phosphorylation of cyclic AMP response element–binding protein (CREB), which is linked to Akt activation^21^ and important for adipogenesis signaling^22^, revealed no major difference between the two MEFs except for a slightly more sustained expression in *Txnrd1*^*fl*/*fl*^ cells at the 120 min time point (Fig. 3C). However, activating transcription factor 1 (ATF1), which is closely related to CREB and shares similar functional motifs^23^, was more phosphorylated in *Txnrd1*^−/−^ MEFs after hormonal induction of differentiation (Fig. 3C). Another effector of insulin action involved in adipogenesis is mTOR1^24^, the activity of which is illustrated by the levels of phosphorylation of its downstream target ribosomal protein S6^25^. Intriguingly, we found that the baseline phosphorylation level of S6 was lower in *Txnrd1*^−/−^ MEFs compared to the parental cells, but more responsive with strongly increased phosphorylation upon induction of differentiation (Fig. 3C). These results collectively demonstrate clearly enhanced intracellular insulin responsiveness after deletion of the *Txnrd1* gene.

Previous studies have shown that the Trx system helps to reduce and thus activate oxidized protein-tyrosine phosphatase 1B (PTP1B) in relation to PDGF signaling^26^. Because PTP1B is an important negative regulator also of insulin signaling, as it dephosphorylates key tyrosine residues of the insulin receptor (IR)^27^, we next assessed IR phosphorylation status. We observed no major difference in phosphorylation of tyrosine residues on IR when comparing parental and *Txnrd1*^−/−^ MEFs after rosiglitazone treatment, with similarly increased phosphorylation of IR in both cell types (Fig. 3D). This suggests that the difference in insulin responsiveness between *Txnrd1*^−/−^ and *Txnrd1*^*fl*/*fl*^ MEFs should be explained by signaling events downstream of IR activation, which was analyzed next.

### *Txnrd1* deletion sensitizes cells to insulin signaling by attenuating PTEN activity

**Figure 4:**
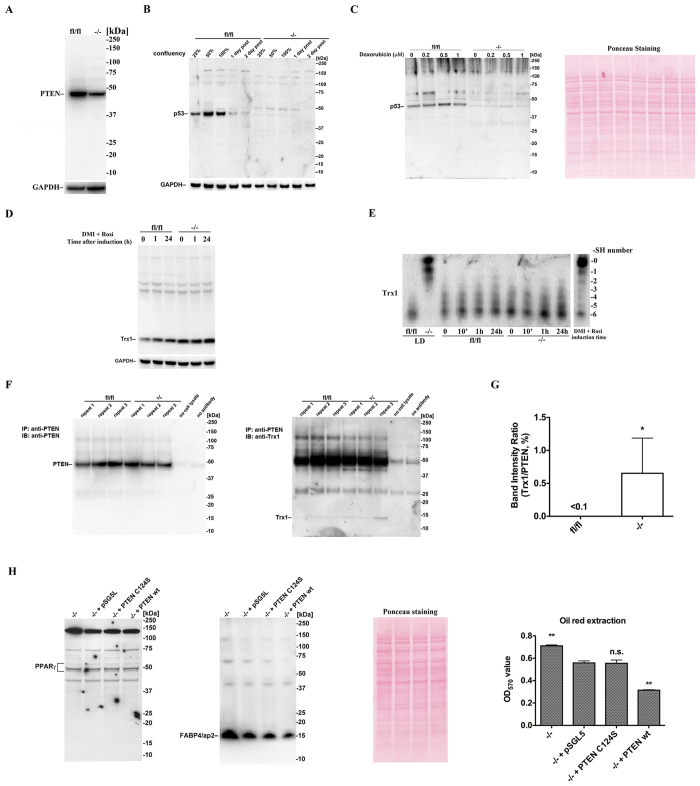
Attenuated cellular PTEN activity contributes to insulin sensitization and adipogenesis in *Txnrd1*^−/−^ MEFs. (**A**) Lysates of *Txnrd1*^*fl*/*fl*^ and *Txnrd1*^−/−^ MEFs were immunoblotted with an antibody against PTEN, using GAPDH as loading control. (**B**) *Txnrd1*^*fl*/*fl*^ and *Txnrd1*^−/−^ MEFs were cultured into different stages of confluency, and lysates were immunoblotted with an antibody against p53, using GAPDH as loading control. (**C**) *Txnrd1*^*fl*/*fl*^ and *Txnrd1*^−/−^ MEFs were cultured to 100% confluency and then treated 24 h with doxorubicin as indicated. Lysates were immunoblotted with an antibody against p53 using Ponceau S staining as loading control. (**D**) *Txnrd1*^*fl*/*fl*^ and *Txnrd1*^−/−^ MEFs were induced for hormonal adipocyte differentiation for the indicated time, and lysates were immunoblotted with an antibody against Trx1. (**E**) *Txnrd1*^*fl*/*fl*^ and *Txnrd1*^−/−^ MEFs were induced to adipocyte differentiation with time points as indicated and the redox state of Trx1 was analyzed by redox immunoblotting^15^. Lysates of *Txnrd1*^*fl*/*fl*^ and *Txnrd1*^−/−^ MEFs cultured at low density (LD, 1000 cells/cm^2^) for 20 h in high glucose medium^15^ were used as negative and positive controls for Trx1 oxidation, respectively. Migration of Trx1 in a standard curve of MEF lysate treated so that Trx1 variants in all forms from 0 to 6 free Cys thiol (-SH) groups being present^70^ is shown in the last lane. (**F**) Lysates of *Txnrd1*^*fl*/*fl*^ and *Txnrd1*^−/−^ MEFs were immunoprecipitated with an antibody against PTEN and then immunoblotted with an antibody against either PTEN (left) or Trx1 (right), with three repeats for each cell line or controls without cell lysate or antibody against PTEN as indicated. (**G**) Band intensities in F were quantified with the ratio between Trx1 and PTEN plotted (n = 3, ±SEM) and significant difference indicated (*P < 0.05). (**H**) Confluent *Txnrd1*^−/−^ MEFs transfected for expression of PTEN variants as indicated were induced with adipocyte differentiation treatment. Lysates were subsequently immunoblotted with antibodies against PPARγ or FABP4/aP2, with Ponceau S staining as loading control. Oil Red-O staining after differentiation was also extracted with color intensity measured (n = 4–8, ±SEM). Significant differences against cells transfected with empty vector pSG5L are indicated (n.s. no significant difference; **P < 0.01).

Insulin signaling requires phosphatidylinositol (PI) 3-kinase (PI3K)-mediated phosphatidylinositol (3,4,5)-trisphosphate (PIP_3_) generation, which in turn activates Akt/PKB and this process is negatively regulated by the phosphatase and tensin homologue (PTEN) protein^20^. Here we found that PTEN protein levels were lower in *Txnrd1*^−/−^ cells compared to the parental *Txnrd1*^*fl*/*fl*^ MEFs (Fig. 4A). Many factors are known to regulate PTEN expression, including p53^28^ that upregulates PTEN at both the mRNA and protein levels^29^,^30^. In mammalian cells, TrxR inhibition was shown to impair p53 conformation and function^31^, which prompted us to analyze p53 levels in our cell models. Interestingly, p53 was hardly detectable in *Txnrd1*^−/−^ MEFs, regardless of stages of cell growth or upon doxorubicin treatment, which normally increases p53 expression. In contrast, p53 levels were clearly responding in *Txnrd1*^*fl*/*fl*^ MEFs with a higher expression during proliferation or upon doxorubicin treatment (Fig. 4B,C). It is thus possible that p53 can contribute to the higher PTEN levels in *Txnrd1*^*fl*/*fl*^ cells compared to *Txnrd1*^−/−^, but it is known that PTEN can also be regulated by several different mechanisms.

It was reported that Trx1 can bind to and inhibit PTEN in a redox dependent manner, which may result in activated Akt^32^, but, conversely, Trx1 can also reduce oxidized PTEN to increase its activity^33^,^34^. We therefore investigated the levels of Trx1 as well as its redox status. We first confirmed that Trx1 protein levels are upregulated in *Txnrd1*^−/−^ MEFs upon hormonal treatment (Fig. 4D), as reported previously for the regularly propagated cells^15^. In our earlier study, we showed that Trx1 was mostly present in its reduced form in *Txnrd1*^−/−^ cells, unless the cells were challenged with exaggerated oxidative stress^15^. Here we found that Trx1 was maintained mostly in its reduced form in *Txnrd1*^−/−^ MEFs also after hormonal induction of adipocyte differentiation (Fig. 4E). Co-immunoprecipitation studies furthermore showed that a physical interaction between Trx1 and PTEN could be detected in *Txnrd1*^−/−^ cells, but not in the parental MEFs (Fig. 4F,G). Taken together, these results would be compatible with Trx1-mediated binding to and inhibition of PTEN in the *Txnrd1*^−/−^ cells, which may further contribute to their sensitization to insulin signaling. To examine this possibility, we transiently overexpressed wildtype (wt) PTEN, or its inactive C124S mutant derivative, in the *Txnrd1*^−/−^ MEFs. This showed that overexpression of wt PTEN counteracted the adipogenesis of *Txnrd1*^−/−^ cells, as illustrated by lower levels of the adipocyte marker FABP4/aP2 and less Oil Red-O stained lipid accumulation, with no major effects on PPARγ levels (Fig. 4H). Collectively, these findings suggest that attenuated PTEN activity due to lower PTEN levels and inhibitory binding of Trx1 should be a part of the molecular mechanisms explaining the augmented adipogenesis observed in *Txnrd1*^−/−^ MEFs.

### NAC supplementation blocks adipogenesis in *Txnrd1* ^−/−^ MEFs

**Figure 5:**
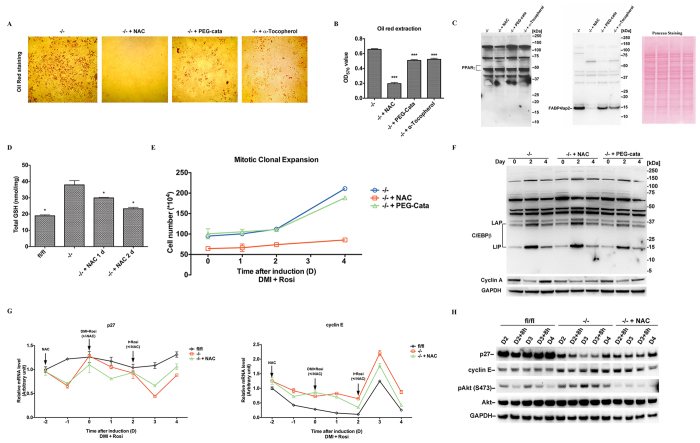
NAC treatment blocks adipocyte differentiation of *Txnrd1*^−/−^ MEFs. (**A**) Confluent *Txnrd1*^−/−^ MEFs were pretreated with 2 mM NAC, 100 U/ml PEG-catalase or 100 μM α-tocopherol for two days, before induction with standard adipocyte differentiation treatment and keeping the antioxidants present throughout the treatment for 8 days. The cells were subsequently stained with Oil Red-O; here 5x magnification was used for imaging. (**B**) The Oil Red-O staining was extracted and the color intensity was measured (n = 8, ±SEM), with significant differences between untreated controls and treated samples indicated (***P < 0.05). (**C**) Lysates of the MEFs in A were immunoblotted with antibodies against PPARγ and FABP4/aP2, with Ponceau S staining used as loading control. (**D**) GSH + GSSG levels (total GSH) were measured in *Txnrd1*^*fl*/*fl*^, untreated or 2 mM NAC treated *Txnrd1*^−/−^ MEFs for time points as indicated (n = 3, ±SEM). Significant differences between untreated *Txnrd1*^−/−^ MEFs and the other samples are indicated (*P < 0.05). (**E**) Confluent *Txnrd1*^−/−^ MEFs were pretreated with 2 mM NAC or 100 U/ml PEG-catalase for two days, before they were induced with adipocyte differentiation treatment with the antioxidants present. Cell numbers at different time points after induction were determined and plotted (n = 4, ±SEM). (**F**) The cell lysates of the different time points in E were immunoblotted with antibodies against C/EBPβ and cyclin A, with GAPDH as loading control. (**G**) Confluent *Txnrd1*^−/−^ cells were incubated with 2 mM NAC for two days and then induced with adipocyte differentiation treatment as described for indicated time points in the NAC treated samples. *Txnrd1*^*fl*/*fl*^ and *Txnrd1*^−/−^ cells not treated with antioxidants were used for comparison. The mRNA levels of *p27* and *cyclin E* at different time points were measured using real time-PCR and plotted by normalizing to *18 S* (n = 3, ±SEM). (**H**) The cell lysates of the MEFs at different time points between day 2 (D2) and day 4 (D4) cultured as in (**G**) were immunoblotted with antibodies against p27, cyclin E, pAkt (S473) and total Akt, with GAPDH used as loading control. See Supplementary Material for non-cropped immunoblots.

Since physiological increases in H_2_O_2_ levels have been proposed as important triggers of adipocyte differentiation^35^ and because we found earlier that *Txnrd1*^−/−^ MEFs present higher levels of H_2_O_2_^15^, we next investigated if supplementation with antioxidants could blunt the increased adipogenesis process in these cells. Indeed, PEG-catalase and α-tocopherol attenuated adipocyte formation and lipid accumulation. Strikingly, N-acetyl cysteine (NAC) supplementation completely abolished adipogenesis in *Txnrd1*^−/−^ MEFs (Fig. 5A,B). None of these treatments affected the expression levels of PPARγ (Fig. 5C). However, in agreement with lower adipogenesis capability (Fig. 5A), the treatments inhibited FABP4/aP2 expression, with NAC totally abrogating its expression (Fig. 5C). We furthermore found that NAC did not increase, but rather decreased, the total glutathione (GSH + GSSG) levels in *Txnrd1*^−/−^ MEFs, although these were still higher than in the parental *Txnrd1*^*fl*/*fl*^ MEFs (Fig. 5D). Thus, the blocking effects of NAC were not necessarily due its function as a cysteine and GSH precursor, and could involve several of the other reported effects of NAC supplementation^36^. We further found that NAC, but not PEG-catalase, almost completely abolished the capacity for MCE of *Txnrd1*^−/−^ MEFs (Fig. 5E), which should be part of the explanation how NAC completely inhibited adipogenesis in these cells. However, neither NAC nor PEG-catalase affected C/EBPβ expression after hormonal induction (Fig. 5F). Upregulation of cyclin A in *Txnrd1*^−/−^ MEFs was however attenuated by NAC treatment, which agrees with its blocking effect on MCE (Fig. 5F).

We next investigated NAC effects on transcription levels of cyclin-dependent kinase inhibitors and cyclins. This revealed that p27, an inhibitor of cell cycle progression^37^, was downregulated during initiation of adipocyte differentiation in *Txnrd1*^−/−^ MEFs compared to parental cells, but that NAC treatment attenuated the fluctuations in its transcript levels at later time points (Fig. 5G). Conversely, the transcript levels of cyclin E were higher in *Txnrd1*^−/−^ MEFs and increased to a larger extent on day 3 compared to *Txnrd1*^*fl*/*fl*^ MEFs, with NAC partially attenuating this difference (Fig. 5G). To further validate these findings, we analyzed the protein levels of p27 and cyclin E at different time points between day 2 and day 4 after hormonal induction of adipogenesis (Fig. 5H). Reflecting the effects on mRNA levels, the p27 protein levels were much lower in *Txnrd1*^−/−^ compared to *Txnrd1*^*fl*/*fl*^ MEFs and further decreased on day 3, whereas NAC treatment increased the p27 protein levels in these cells (Fig. 5H). Cyclin E was also higher in the knockout MEFs compared to parental cells, with NAC slightly lowering its expression towards the later time points (Fig. 5H). Akt activation has been reported to depress, both directly and indirectly, the expression levels of p27^38^,^39^. Here we indeed found that Akt was more activated in the knockout cells, as indicated by phosphorylation of its Ser473 residue, while NAC prevented this activation (Fig. 5H).

### Extensive activation of PPARγ in *Txnrd1* ^−/−^ MEFs

**Figure 6:**
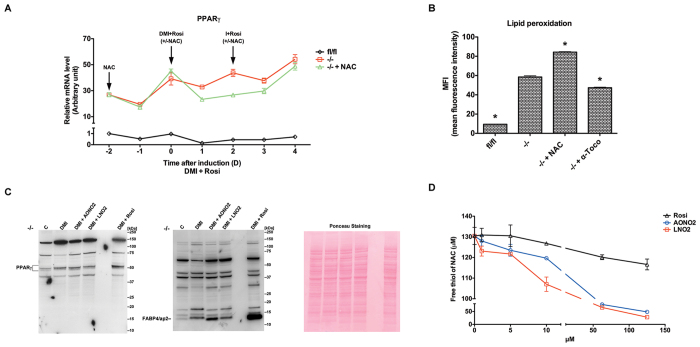
Extensive upregulation of PPARγ expression in *Txnrd1*^−/−^ MEFs. (**A**) The mRNA levels of PPARγ in Fig. 4G were measured using real time-PCR and plotted as normalized to 18 S (n = 3, ±SEM). (**B**) Lipid peroxidation in *Txnrd1*^*fl*/*fl*^, *Txnrd1*^−/−^ and *Txnrd1*^−/−^ MEFs treated with 100 μM α-tocopherol or 2 mM NAC for two days was assessed using Bodipy (581/591) staining. Bodipy oxidation was analyzed by FACScan in the FITC channel. Mean fluorescence intensity (MFI) was plotted (n = 3–4, ±SEM), with significant differences between untreated *Txnrd1*^−/−^ MEFs and the other samples indicated (*P < 0.05). (**C**) *Txnrd1*^−/−^ MEFs were induced to adipocyte differentiation using DMI, DMI plus 3 μM AONO_2_, LNO_2_ or Rosiglitazone respectively as described for 8 days. The cell lysates were subsequently immunoblotted with antibodies against PPARγ or FABP4/aP2, with Ponceau S staining used as loading control. (**D**) 130 μM NAC was incubated with the indicated concentration of AONO_2_, LNO_2_ or Rosiglitazone respectively for 30 min. Then, the level of free thiols of NAC was detected by adding DTNB and measuring the absorbance at 412 nm (n = 3, ±SEM).

We next found that *Txnrd1*^−/−^ MEFs displayed as much as 30-fold higher basal PPARγ expression levels than the parental cells, which were further elevated upon adipocyte differentiation. NAC treatment somewhat inhibited upregulation of PPARγ levels in the initial days of the differentiation process, but not nearly down to the levels found in the parental cells (Fig. 6A). Not only PPARγ expression levels but also extent of activation can thereby contribute to the observed phenotypes, which we studied next.

Nitration modified unsaturated fatty acids are endogenous PPARγ ligands that can be induced upon oxidative stress^40^,^41^. We thus next assessed lipid peroxidation levels in our cell models using a Bodipy (581/591) reagent. *Txnrd1*^−/−^ MEFs had six times higher levels of lipid peroxides compared to *Txnrd1*^*fl*/*fl*^ MEFs, which were to some extent dampened by treatment with the lipid soluble antioxidant α-tocopherol. Treatment with NAC, in contrast, failed to reduce lipid peroxidation in the knockout MEFs but instead increased the signal (Fig. 6B). These findings suggested that the very high expression of PPARγ together with higher lipid peroxide levels could provide a mechanism for increased adipogenic potential of *Txnrd1*^−/−^ MEFs, but the potential involvement of nitrated lipids and their possible prevention by NAC was still unclear.

To investigate direct effects of nitro-modified unsaturated fatty acids, we attempted to stimulate differentiation of the *Txnrd1*^−/−^ MEFs using either 9-Nitrooleate (AONO_2_) or 10-Nitrolinoleate (LNO_2_) instead of using the standard PPARγ ligand rosiglitazone in the hormonal induction cocktail. Interestingly, treatment of *Txnrd1*^−/−^ MEFs with dexamethasone, methylisobutylxanthine and insulin alone (DMI) increased expression of PPARγ and FABP4/aP2, while addition of AONO_2_, but not of LNO_2_, further promoted adipocyte formation as illustrated by increased FABP4/aP2 expression (Fig. 6C). Notably, neither of these two compounds upregulated the PPARγ expression levels more than DMI treatment alone (Fig. 6C). These findings agree with the notion that AONO_2_ directly activates PPARγ as a ligand^40^. Because nitro-modified unsaturated fatty acids are highly electrophilic and can interact directly with free thiols^42^ we asked whether NAC, having a readily accessible thiol group, might directly scavenge these electrophilic metabolites. If so, that could potentially block adipocyte differentiation as a result of inhibited PPARγ activation. Indeed, both AONO_2_ and LNO_2_, but not rosiglitazone, were found to readily react with the free thiol group of NAC at roughly stoichiometric amounts upon 30 min incubation *in vitro* (Fig. 6D). If such direct scavenging would occur also in of *Txnrd1*^−/−^ MEFs, this should likely contribute to the antiadipogenic effect of NAC treatment.

### *TXNRD1* knockdown promotes adipogenesis of human primary cells

**Figure 7:**
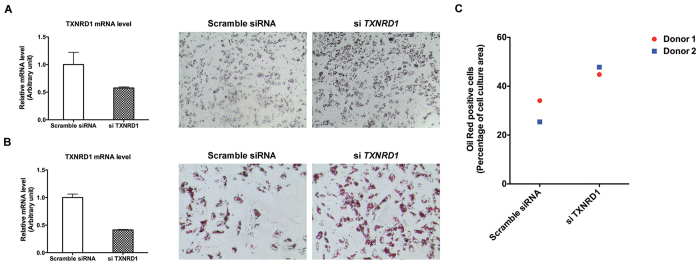
*TXNRD1* knockdown promotes adipogenesis in human pre-adipocytes. (**A**) Left panel, pre-adipocytes isolated from donor 1 were cultured with additional of 50 nM selenite, transfected with scramble siRNA or with siRNA against *TXNRD1*, with mRNA levels of *TXNRD1* in these cells measured using real time-PCR and plotted as normalized to LRP10 (n = 3, ±SEM). Right panel, the same treated cells were induced for adipocyte differentiation for 6 days. Thereafter, the PPARγ agonist was removed from the medium and the cells were incubated for additional 5 days before Oil Red-O staining; 4x magnification was used for imaging. (**B**) Pre-adipocytes isolated from donor 2 were plated without selenite supplementation, and subsequently treated and analyzed the same way as described in A; 10x magnification was used for imaging. (**C**) Oil Red-O staining in (**A**,**B**) plotted upon quantification using the Image J software.

To probe if TrxR1 expression counteracts adipogenesis also in primary human cells, we isolated stroma vascular cells from abdominal subcutaneous adipose tissue that were subsequently cultured and induced to differentiate *in vitro*. Following hormonal induction (DMI + Rosi), we found that cells treated with siRNA-mediated *TXNRD1* knockdown significantly increased their adipogenesis, as demonstrated by a stronger Oil Red-O staining, compared to cells that had been transfected with non-silencing siRNA. This was seen with cells from two different donors and either with or without selenium supplementation to the growth medium (Fig. 7A–C).

### *TXNRD1* transcript levels associate with human insulin resistance *in vivo*

To assess whether *TXNRD1* expression in adipose tissue could be related to clinical and/or adipocyte-specific aspects of insulin sensitivity, we analyzed data from a global transcriptional profiling study in 56 women (30 obese and 26 non-obese)^43^. We found that *TXNRD1* levels correlated with body mass index (BMI, r = 0.56, p < 0.0001). Thus, to avoid a bias for BMI as a confounding factor, multiple regression analyses were performed. This revealed that *TXNRD1* transcript levels in adipose tissue were inversely associated with *in vivo* measures of insulin responsiveness (determined with an intravenous insulin tolerance and plasma glucose reduction test), as well as adipocyte responsiveness to insulin *in vitro* (determined as insulin-stimulated lipogenesis at maximal effective concentration). Notably, these relationships were specific for insulin responsiveness as there were no BMI-independent correlations between *TXNRD1* and other measures of lipid or adipocyte metabolism such as e.g. plasma triglyceride levels or adiponectin secretion (Table 1). These findings revealed that, similarly to our findings in the MEF cell models, *TXNRD1* expression levels correlated with insulin responsiveness and lipogenesis also in this human cohort.

## Discussion

**Figure 8:**
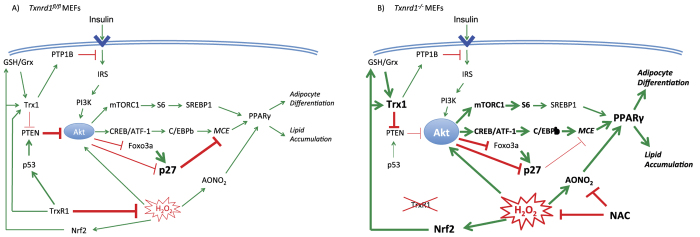
Signaling mechanisms triggering adipogenesis in MEFs lacking *Txnrd1*. These schemes graphically summarize our findings as reported herein regarding signaling pathways in (**A**) *Txnrd1*^*fl*/*fl*^ functionally wildtype MEFs compared to (**B**) *Txnrd1*^−/−^ knockout cells that contribute to increased adipogenesis in the latter. Stimulatory signaling pathways are indicated with green arrows while inhibitory mechanisms are shown with red block arrows. Note that many of these pathways may be indirect, rather than direct as simplified in these schemes. Thicker arrows and bold font suggest increased activities. Processes (rather than proteins or molecules) are indicated in italics, with *MCE* being “mitotic clonal expansion”. Note the increased Akt activity in *Txnrd1*^−/−^ MEFs that together with PPARγ activation and increased oxidative stress (indicated by H_2_O_2_ and AONO_2_) triggers increased adipogenesis, which may be counteracted by NAC treatment. The mechanisms indicated in these schemes are further discussed in the text.

We recently found that MEFs lacking TrxR1 (*Txnrd1*^−/−^) undergo massive cell death when cultured at low density (<1000 cells/cm^2^) in high-glucose medium, due to impaired self-sufficient growth and insufficient elimination of glucose-derived H_2_O_2_^15^. However, when cultured at higher density (>8000 cells/cm^2^) these cells grow well due to a release of catalase activity to the medium sufficient to detoxify their higher levels of metabolism-derived H_2_O_2_^15^. *Txnrd1*^−/−^ cells could thus be utilized herein as a well-defined cell culture model system for in-depth investigations concerning metabolic consequences of TrxR1 removal. Here we demonstrated that *Txnrd1* deletion channels glucose usage towards increased basal mitochondrial respiration and anabolic pathways, resulting in increased glycogen and lipid biosynthesis. The differences in basal mitochondrial respiration but not in spare respiratory capacity could be due to effects on respiratory complex assembly or perhaps redox-derived post-translational modifications of respiratory subunits. Interestingly, recent evidence has shown how oxidative stress can trigger signaling pathways that modulate respiratory rates^44^. Our results clearly warrant further research to delineate the role of TrxR1 in regulating mitochondrial respiration. We also showed that deletion or knockdown of TrxR1 strongly improves the capacity of immortalized MEFs or human pluripotent primary cells to undergo adipogenesis. The highly significant inverse association between *TXNRD1* transcript levels in human adipose tissue and insulin responsiveness *in vivo* and *in vitro* suggests that our findings have relevance for human physiology. From our more detailed analyses of *Txnrd1*^−/−^ MEFs, insights with regards to the molecular mechanisms involved could be gained. These mechanisms are graphically summarized in Fig. 8 and discussed further as follows.

Insulin-triggered intracellular signaling cascades are known to play essential roles in adipocyte differentiation^3^. Our findings of stronger activation of Akt and CREB/ATF1 in the *Txnrd1*^−/−^ MEFs correlate well with a more pronounced adipocyte differentiation phenotype being accentuated by exaggerated insulin signaling. As the phosphotyrosine phosphatase PTP1B is known to be an important negative regulator of insulin signaling by catalyzing dephosphorylation of IR^27^, and because PTP1B is reduced and activated by the TrxR1-dependent Trx system^26^, we were initially surprised not to detect any significant differences in IR phosphorylation status between *Txnrd1*^−/−^ and *Txnrd1*^*fl*/*fl*^ MEFs. One explanation might be that in the previous study where the Trx system was found to act in PTP1B activation, *Txnrd1*^−/−^ MEFs were starved for 24 h using 1% FBS and thereupon induced with platelet-derived growth factor (PDGF)^26^. Starvation is well known to induce oxidative stress and may thus have exaggerated PTP1B oxidation in the absence of TrxR1. Also, here we studied IR status and not the PDGF receptor and the exact ligand-receptor mediated phosphorylation cascades where the Trx system-catalyzed activation of PTP1B is important need to be further studied. Our findings in the present study however strongly suggest that the increased capacity for adipocyte differentiation of MEFs upon *Txnrd1* deletion should be explained by accumulated effects of several altered signaling events downstream of IR that lead to increased insulin responsiveness. Such effects are likely to contribute to the inverse correlation between *TXNRD1* transcription and clinical parameters of insulin action as found in our human cohort of obese and non-obese women, even though detailed mechanistic studies must be performed also with human cells and tissues before such assumptions can be fully confirmed.

Our finding that the expression level of the negative Akt regulator PTEN was lower in the TrxR1 deficient MEFs should be important for the observed phenotype, with the PI3K/Akt axis being crucial for insulin triggered adipogenesis^45^. Further studies are needed to validate whether the low p53 levels in *Txnrd1*^−/−^ cells are an important factor explaining their propensity to differentiate. That would fit with earlier observations that p53 can be an efficient suppressor of white adipocyte differentiation^46^.

The interplay between the Trx system and regulation of PTEN activity is likely to be complex. PTEN is a redox sensitive phosphatase, which becomes inactivated upon exposure to H_2_O_2_ and formation of a disulfide bond between Cys71 and Cys124^33^,^34^. Trx1 has been shown to reduce and reactivate this type of oxidized PTEN^33^,^34^. However, another study found that the reduced form of Trx1 directly binds to PTEN to inhibit its activity^32^. We showed previously, and also here, that the Trx1 protein becomes upregulated in *Txnrd1*^−/−^ MEFs, which can at least in part be due to Nrf2 activation^15^. Importantly, here we show that Trx1 can be maintained in its reduced form also in the absence of TrxR1 and regardless of the state of adipocyte differentiation. Since GSH and Grx can constitute a backup system for Trx1 reduction when TrxR1 becomes impaired^14^,^47^,^48^, especially with the GSH-dependent enzyme systems being upregulated in the *Txnrd1*^−/−^ MEFs^15^, this results in the apparent paradox that the levels of reduced Trx1 can become higher than normal in TrxR1 knockout cells. As our immunoprecipitation experiments suggested a stronger interaction between Trx1 and PTEN in *Txnrd1*^−/−^ cells, this would be compatible with the notion of Trx1 binding to and serving as an inhibitor of PTEN^32^. The attenuated adipocyte differentiation seen upon overexpressed PTEN in the *Txnrd1*^−/−^ MEFs further supports this model.

Primary MEFs are normally able to differentiate *in vitro* upon induction using treatment with pro-adipogenic factors^3^,^49^, whereas most immortalized MEFs have lost the capacity to differentiate^3^,^49^. Here we showed that the basal levels of TrxR activity in the parental immortalized *Txnrd1*^*fl*/*fl*^ MEFs are two-fold higher than in primary MEFs. Upon long time treatment with proadipogenic factors, primary MEFs could differentiate into the adipocyte lineage, but the immortalised *Txnrd1*^*fl*/*fl*^ MEFs could not, consistent with our findings that TrxR1 suppresses adipocyte differentiation. With *Txnrd1* depletion restoring and enhancing the adipocyte differentiation capacity of immortalised MEFs, this became a vivid illustration of the importance of TrxR1 for regulation of adipogenesis in MEFs. Interestingly, the *Txnrd1*^−/−^ MEFs seemed to differentiate into brown-like or beige adipocytes, as reflected by increased UCP1 expression^3^,^50^. Thus, TrxR1 may possibly also play specific roles in regulation of white *vs* beige adipocyte differentiation, which should clearly be studied further.

To understand the mechanisms underpinning the increased adipogenesis in *Txnrd1*^−/−^ MEFs, it is important to note that MCE is considered essential for adipogenesis of MEFs and 3T3-L1 cells^17^,^18^. As the *Txnrd1*^*fl*/*fl*^ MEFs were unable to undergo MCE, this could be a main reason for their inability to differentiate into adipocytes. It was in this context interesting that TrxR1 deficient MEFs spontaneously underwent MCE, with hormonal inducers further augmenting the process. As shown before, and also found here with regards to lipid peroxidation, depletion of *Txnrd1* in MEFs leads to a heightened oxidative stress^15^. Increased production of H_2_O_2_ can be detrimental if excessive, but can facilitate adipocyte differentiation through enhancement of signal transduction and acceleration of MCE^35^,^51^,^52^. Increased H_2_O_2_ levels helping to trigger adipogenesis is compatible with our findings that supplementation with antioxidants inhibited adipocyte formation. It was particularly intriguing that NAC completely abrogated adipocyte differentiation, partially through blocking of the MCE. One possible part of that effect could involve increased p27 levels, as p27 is well known to be a crucial protein regulated by oxidative events and causing cell cycle arrest^53^,^54^. Downregulation of p27 is also tightly connected to Akt activation^39^,^55^, which agrees with our findings in the *Txnrd1*^−/−^ MEFs. Activated Akt phosphorylates p27 at threonine 157, which localizes p27 to the cytosol and leads to proteasomal degradation^39^,^56^. Akt also phosphorylates FOXO3a and inhibits its transactivation of p27 expression^55^. As NAC treatment blocked Akt activation in the knockout cells, it appears that oxidative stress can contribute to Akt activation in these cells, as shown earlier in other cell types^57^,^58^.

The importance of PPARγ in relation to our findings should be its ligand-mediated activation and pluripotent roles in adipogenesis^59^. Nitro-modified unsaturated fatty acids are endogenous PPARγ ligands acting at sub-micromolar concentration^40^, of which the production becomes facilitated by increased oxidative stress^41^. As we detected higher lipid peroxidation levels in the *Txnrd1*^−/−^ MEFs compared to the *Txnrd1*^*fl*/*fl*^ cells, this suggests that more nitro-modified unsaturated fatty acids are indeed produced in the knockout cells, which subsequently may activate PPARγ. Indeed, DMI treatment without inclusion of the PPARγ ligand rosiglitazone could still activate PPARγ and upregulate FABP4/aP2 expression in those cells, indicating that there must be an endogenously increased PPARγ ligand production in *Txnrd1*^−/−^ cells. Since we further found that addition of AONO_2_ could promote adipocyte formation and that NAC could bind nitrated fatty acids directly *in vitro*, such scavenging may well have been another mechanism to explain the complete NAC blockage of the adipocyte differentiation that we observed.

In conclusion, our findings demonstrate that depletion of *Txnrd1* promotes adipocyte differentiation of immortalized MEFs. This occurs through enhanced responsiveness to insulin signaling, with attenuated PTEN activity, loss of cell cycle control by activation of Akt, downregulation of p27, and activation of PPARγ. Our observations that *TXNRD1* transcript levels inversely associate with clinical signs of human insulin resistance *in vivo* and in adipocytes *in vitro* warrant further studies addressing the potential role of TrxR1 as a therapeutic target in diseases affecting glucose and/or lipid metabolism. As suggested here, TrxR1 is as important modulator of cell fate and metabolism through suppression of insulin signaling and adipocyte differentiation.

## Materials and Methods

### Materials

The expression plasmid encoding wildtype (wt) PTEN (catalog number 10750) and mutant C124S PTEN (10744) with the control empty plasmid were all from Addgene (Cambridge, MA, USA) and have been described elsewhere^60^. 9-Nitrooleate (AONO_2_) and 10-Nitrolinoleate (LNO_2_) were purchased from Cayman Chemical (Ann Arbor, MI, USA). All chemicals were obtained from Sigma-Aldrich Chemicals (St. Louis, MO, USA) unless stated otherwise.

### Ethics

Studies in humans were approved by the regional ethical review board of Stockholm (*Regionala Etikprövningsnämnden i Stockholm*) and were conducted in full accordance with the Declaration of Helsinki. Informed written consent was obtained from all participants. Patient cells were obtained under ethical permit nr. 326/03 and array analyses were performed under ethical permit nr. 2011/1002-31/3. The mouse embryonic fibroblasts (MEFs) were isolated in Munich, Germany, in accordance with the German Animal Welfare Law and approved by the institutional committee on animal experimentation and the government of Upper Bavaria, Germany, under statements nr. VII 7/8730-1/6/99 and 211-2531.1-19/04.

### MEFs cell cultures and adipocyte differentiation

The establishment of wildtype TrxR1 expressing parental MEFs having exon 15 of the *Txnrd1* gene flanked by flox sites (*Txnrd1*^*fl*/*fl*^) and the full TrxR1 knockout MEFs derived from *Txnrd1*^*fl*/*fl*^ cells after Cre treatment *in vitro* (hereafter referred to as *Txnrd1*^−/−^) were previously described^15^. The MEFs were kept at >10% confluence (split 1:8 or less for culture maintenance) in DMEM with 4.5 g/l glucose content, supplemented with 4 mM glutamine, 100 U/ml penicillin, 100 mg/ml streptomycin (BioWhittaker, Lonza, NJ, USA) and 10% (v/v) fetal bovine serum (PAA Laboratories, Piscataway, NJ, USA). No extra selenium source was added unless mentioned. Cells were grown in humidified air containing 5% CO_2_ at 37 °C for all experiments. Primary MEFs were isolated from pregnant mice at day 13.5 post coitum. Single cells were obtained by shaking (100 rpm) for 15 minutes at 37 °C with 0.05% trypsin/EDTA (Invitrogen), DNase (100 U/ml, Roche) and glass pearls. The reaction was stopped by adding medium with 10% FCS. Cells were then cultured in DMEM medium mentioned above with 0.2% gelatin-coated flask. To induce differentiation, 2-day postconfluent MEFs (designated day 0) were cultured in DMEM containing 10% FBS, 1 μM dexamethasone, 0.5 mM methylisobutylxanthine, 5 μg/ml insulin and 0.5 μM rosiglitazone, unless stated otherwise. From day 2, medium containing 5 μg/ml insulin and 0.5 μM rosiglitazone was changed every other day until day 8 or as indicated.

### *TXNRD1* knockdown and adipocyte differentiation in primary human pre-adipocytes

Human adipose tissue was obtained from adult human subjects undergoing surgery for non-malignant conditions. Although both donors were anonymous to us, they had given informed written consent to donate tissue that would have otherwise been discarded. The study was approved by the regional board of ethics. Human adipose tissue-derived stromal vascular cells were isolated as previously described^61^ and cultured in 12-well format as previously described^62^ with one exception; differentiation was induced 48 h instead of 24 h after plating in order to downregulate *TXNRD1* prior to initiation of adipogenesis. Gene expression of *TXNRD1*, which encodes TrxR1 in human, was knocked down using siRNA (sense 5′-(GCAAGACUCUCGAAAUUAU)dTdT-3′, antisense 5′-(AUAAUUUCGAGAGUCUUGC)dAdG-3′). Control cells were transfected with Allstars negative control (Qiagen, Hilden, Germany). In all cells, transfections were performed using HiPerFect transfection reagent (Qiagen, Hilden, Germany) with 20 nM siRNA at two time points, 24 h and 48 h after plating. Differentiated cells were stained with Oil Red-O as described below 12–14 days after plating.

### Oil Red-O staining

Cells were washed three times with PBS (Phosphate-buffered saline) and then fixed with 4% formaldehyde for 1 h. Oil Red-O (0.3% in isopropanol) was diluted with water (3:2), filtered through a 0.22 μm filter, and incubated with the fixed cells for 30 min at room temperature. Cells were then washed with water, whereupon stained neutral fat droplets were visualized by light microscopy and photographed. Oil Red-O was thereafter extracted using isopropanol and absorbance was measured at 570 nm for quantification.

### Triglyceride measurement

The cells were seeded on 150 mm dishes and cultured for 4 days to reach confluence. Triglyceride contents were then measured using a Triglyceride Quantification Kit (ab65336, Abcam, Cambridge, UK) according to the instruction manual.

### Glycogen measurement

The cells were seeded on 150 mm dish and cultured for 4 days to reach confluence. Glycogen contents were measured using a Glycogen Assay Kit II (#ab169558; Abcam, Cambridge, MA, USA) according to the manufacturer’s guidelines.

### Lactate production

The assessment of lactate production as an indication of glycolytic flux was performed as previously described with slight modifications^63^. Cells were seeded in M12 well plates, at a density of 50× 10E5 cells per well. The day after, the cells were changed to DMEM containing 2% FBS and 5 mM glucose or 25 mM glucose. Then 50 ul of media were collected at 4 h, 6 h and 8 h for analysis. At the end of the experiment, the cells were lysed (see protocol below for cell lysis) and the total amount of protein was determined for normalization. The amount of lactate present in the media was determined using the LACT (Lactate) reagent from Beckman Coulter (Brea, CA, USA), according to the manufacturer’s guidelines. An additional well per condition was ran in parallel with the glycolytic competitive inhibitor 2-deoxyglucose (30 mM; Sigma-Aldrich, St. Louis, MO, USA). The amount of lactate generated in the presence of 2-deoxyglucose represents the non glycolytic-derived lactate, and was therefore substracted from the overall lactate production. The depicted results represent glycolysis-derived lactate production.

### Measurement of cellular oxygen consumption

Oxygen consumption rate (OCR) in real time on MEFs was performed using an Extracellular Flux Analyzer X24-3 (Seahorse Bioscience, Billerica, MA, USA) as previously described^63^. Cells were seeded at a density of 50x 10E3 cells/well. For the experiment the cells were changed to bicarbonate- and FBS-free DMEM medium (Sigma). The sequential addition of the mitochondrial ATP synthase inhibitor oligomycin (1 uM; EMD Millipore, Billerica, MA, USA), the uncoupling ionophore carbonyl cyanide 4-trifluoromethoxyphenylhydrazone (FCCP; 0.5 uM; Sigma-Aldrich) and the mitochondrial complex III inhibitor antimycin A (5 uM; Sigma-Aldrich) allowed us to determine the fraction of respiration coupled to ATP production, the maximal respiratory capacity and the unproductive respiration not coupled to ATP production, also known as “leak”^64^.

### Analysis of mitochondrial mass in MEFs

*Txnrd1*^*fl*/*fl*^ and *Txnrd1*^−/−^ MEFs were seeded onto M24 wells and cultured for 1 day. The cells were then incubated with 500 nM of MitoTracker Green (Life Technologies, Grand Island, NY, USA) for 15 min at 37 °C, washed twice with culture medium, and visualized using a Zeiss Axiovert 40 CFL fluorescent microscope (Zeiss, Overkochen, Germany), photographed with a coupled Zeiss Axiovert ICm1 camera. Fluorescence intensity was analyzed using the AxioVs40 v4.8.2.0 (Zeiss) and ImageJ v1.48 softwares. All samples were processed in parallel and images captured in the same session using the same imaging parameters. At least 500 cells per well and 3 wells per genotype were quantified for analysis. Data were expressed as Relative Fluorescent Units.

### Glucose uptake

Cells were seeded at a density of 1.5 × 10^5^ cells/well in M6-well plates, and they were allowed to recover over night. Cells were starved for 5 hours in DMEM without glucose, glutamine, pyruvate nor FBS for 5 hours. The medium was then replaced by fresh medium containing 5 ug/ml insulin, and the cells were incubated for 1 hour in this medium. The medium was then replaced with 1 ml of medium per well containing 5 μg/ml insulin, 10 μM 2-deoxyglycose (Sigma-Aldrich, St. Louis, MO, USA) and 1 μCi (20 nM) ^3^H-2-deoxyglucose (American Radiolabeled Chemicals, Inc., St. Louis, MO, USA). Cytochalasin B (Sigma-Aldrich) was used to inhibit insulin-independent glucose transport. Cells were allowed to uptake labeled 2-deoxyglucose for select periods of time (10, 20 and 30 minutes), and then they were extensively washed with chilled PBS and lysed in ice-cold 0.4 N NaOH. The lysates were mixed with Emulsifier Safe Scintillation liquid (Perkin-Elmer, Waltham, MA, USA) and 2-deoxyglucose uptake was quantified using a scintillation counter. Data were determined as counts per minute (CPM) and calculated in 2-deoxyglucose concentration using a known standard of ^3^H-2-deoxyglucose, and normalized by total amount of protein.

### Antibodies

Antibodies against PPARγ (E-8), C/EBPα (14AA), C/EBPβ (C-19), insulin Rβ (C–19), p-Tyr (PY99), p27 (F-8), cyclin A (H-432), cyclin E (M-20), and GAPDH (FL-335) were from Santa Cruz Biotechnologies, Inc. (CA, USA). Antibody against FABP4/aP2 (ab66682) was obtained from Abcam Inc. (Cambridge, MA, USA). Antibodies against phospho-Akt (Thr308) (9275), Phospho-Akt (Ser473) (9271), Akt (9272), phospho-CREB (Ser133) (9191), phospho-S6 (Ser235/Ser236) (4858), S6 (2217), PTEN (9552) came from Cell Signaling Technology, Inc. (Danvers, Ma, USA). The antibodies against β-Actin (#A5441) and Voltage-Dependent Anion Channel-1 (VDAC1; #PC548) were purchased from Sigma-Aldrich (St. Louis, MO, USA) and EMD-Millipore, respectively. Anti-mouse Trx1 antibody serum was a kind gift from Dr. Gary Merrill (Oregon State University, Corvallis, OR, USA).

### Enzyme activity assays

Cellular TrxR activity was measured using end-point insulin reduction assay. 4 μg total cellular protein was incubated with 20 μM recombinant human wt Trx, 297 μM insulin, 1.3 mM NADPH, 85 mM Hepes buffer, pH 7.6, and 13 mM EDTA for 80 min at 37 °C, in a total volume of 50 μl. The reaction was then terminated by adding 200 μl of 7.2 M guanidine-HCl in 0.2 M Tris-HCl, pH 8.0, containing 1 mM DTNB. The extent of Trx-dependent formed thiols in the reduced insulin was then determined by measuring absorbance at 412 nm (extinction coefficient 13,600 M^−1^ cm^−1^) and subtracting a background absorbance for each sample incubated and treated in the same manner containing all components except Trx.

### Immunoblot

Cells were harvested and lysed in RIPA buffer (50 mM Tris-HCl, pH 7.4, 150 mM NaCl, 1 mM EDTA, 1 mm EGTA, 1% Triton X-100, 1% sodium deoxycholic acid, 0.1% SDS, 0.5 mM PMSF, 1.2 mg/ml protease inhibitor cocktail (Roche, Basel, Switzerland), phosphatase inhibitor cocktail 3 (Sigma-Aldrich). The supernatants clarified after centrifugation (13300 r.p.m., 15 min) were used and total protein concentrations were determined with a Bradford reagent kit (Bio-Rad, Hercules, CA, USA). Protein lysates were subjected to SDS/PAGE and transferred onto nitrocellulose membrane using iBlot Dry Blotting System (Thermo Fisher Scientific, Waltham, MA, USA). For detection, the SuperSignal West Pico kit (Thermo Fisher Scientific, Waltham, MA, USA) was used according to the manufacturer’s instructions, with signals detected utilizing a Bio-Rad ChemiDoc XRS scanner and the Quantity One 4.6.7 software.

### Immunoprecipitation

Cells were harvested and lysed in RIPA buffer as described above. First the primary rabbit antibody (2 μg) was pre-incubated with 50 μl of Dynabeads sheep anti rabbit IgG (Life Technologies, Grand Island, NY, USA) with gentle shaking for 3 hours at 4 °C. Total cell lysate (1 to 2 mg protein) was then added into the mixture and incubated with gentle rocking overnight at 4 °C. After several washes of the beads in PBS containing 0.1% BSA and 2 mM EDTA, loading buffer was added to the samples and then they were boiled. Finally proteins in the supernatants were separated on 12% SDS–PAGE, transferred and probed. TrueBlot anti-rabbit IgG (Rockland Immunochemicals, Gilbertsville, PA, USA) was here used as secondary antibody.

### Quantitative Real time-PCR

Total RNA was isolated using the RNAeasy mini kit (Qiagen, Limburg, Netherlands) according to the manufacturer’s protocol. Total RNA (2 μg) was reverse transcribed using Maxima First Strand cDNA synthesis kit (Thermo Fisher Scientific, Waltham, MA, USA) and random hexamer primers. QRT-PCR was performed using Maxima SYBR Green qPCR Master Mix (Thermo Fisher Scientific, Waltham, MA, USA) on PikoReal 96 real time PCR system (Thermo Fisher Scientific, Waltham, MA, USA). The readouts for the mRNA levels of specific genes were normalized to 18S as housekeeping control. Specific primers were designed and synthesized (Thermo Fisher Scientific, Waltham, MA, USA) as follows: mouse p27: sense, 5′-CAGCTTGCCCGAGTTCTACT-3′; antisense, 5′-GAGTTTGCCTGAGACCCAAT-3′; mouse cyclin E: sense, 5′-CCCTTAAGTGGCGTCTAAGC-3′; antisense, 5′-TACTGAGGCATCAGCACCTC-3′; mouse PPARγ: sense, 5′-ATCTTAACTGCCGGATCCAC-3′, antisense, 5′-GATGGCATTGTGAGACATCC-3′; mouse 18S: sense, 5′-ACCGCAGCTAGGAATAATGGA-3′; antisense, 5′-GCCTCAGTTCCGAAAACCA-3′; human TrxR1: sense, 5′-ATATGGCAAGAAGGTGATGGTCC-3′; antisense, 5′-GGGCTTGTCCTAACAAAGCTG-3′; human LRP10: 5′-sense GATGGAGGCTGAGATTGTG-3′; antisense, 5′-GAGTCATATCCTGGCGTAAG-3′.

### Free thiol determination

NAC (130 μM) was incubated with different concentrations of AONO_2_, LNO_2_ or Rosiglitazone as indicated for 30 min in PBS buffer in a 96 well plate. Then free thiols of NAC were quantified by adding 2 mM DTNB and absorbance was measured at 412 nm. The amount of free thiol was calculated using Beer-Lambert law and 14,150 M^−1^ cm^−1^ was used as the extinction coefficiency of TNB^− ^65.

### Assessment of lipid peroxidation

*Txnrd1*^*fl*/*fl*^ and *Txnrd1*^−/−^ MEFs were cultured to confluence, with the *Txnrd1*^−/−^ MEFs incubated with or without 100 μM α-tocopherol for two days. The cells were then stained with 5 μM Bodipy (581/591) at 37 °C for 30 min. Cells were collected and fixed with 0.5% paraformaldehyde in PBS. Signals were analyzed using FACScan with the FITC channel and a total of 10000 cells of each sample were counted.

### Redox immunoblotting of Trx1

Redox immunoblotting was performed based on an electrophoretic mobility-shift assay^47^. Briefly, the cells were lysed with a sample solution (50 mM Tris–HCl, 1 mM EDTA, 8 M urea, pH 8.3) containing 30 mM iodoacetic acid (IAA). After incubation at 37 °C for 30 min, the proteins were precipitated and washed with ice-cold acetone/1 M HCl (98/2 v/v) three times. Then the precipitate was resuspended in the sample solution containing 3.5 mM dithiothreitol (DTT) and incubated at 37 °C for 30 min. Subsequently, the proteins were alkylated with 10 mM iodoacetamide (IAM) and separated by PAGE in buffer containing 8 M urea and transferred onto nitrocellulose membrane using iBlot Dry Blotting System (Thermo Fisher Scientific, Waltham, MA, USA). Trx1 was detected using immunoblotting with antibodies against mouse Trx1. Form of Trx1 having more free thiols became more negatively charged by alkylation with IAA and thus migrated faster.

### ATP Levels

Cells were seeded at a density of 10^4^ cells/well in M96-well plates, and ATP levels were determined using the Cell Titer Glo reagent from Promega (Madison, WI, USA), according to the guidelines provided by the manufacturer, and expressed as relative luminescence units (RLU) and normalized by total amount of protein.

### Human Data

Data on *TXNRD1* expression in subcutaneous abdominal adipose tissue was retrieved from a previously described cohort of 26 non-obese and 30 obese women analyzed by gene micro-array (Human Gene 1.0 ST Array, Affymetrix Inc., Santa Clara, CA)^43^. Global transcription profiling data have been described and are deposited at the National Center for Biotechnology Information Gene Expression Omnibus (http://ncbi.nim.nih.gov/geo) under the accession number GSE25402^43^.

Insulin sensitivity *in vivo* was determined by a short insulin tolerance test as previously described^66^. Briefly, plasma glucose levels after intravenous insulin injection were determined and plotted in a semilogaritmic graph. The rate constant (k) derived from this plot represented the intravenous insulin tolerance (KITT) and was expressed as the % fall in glucose/min between 4 and 16 minutes. Plasma triglycerides (TGs) were determined by the hospital’s accredited clinical chemistry laboratory.

Lipogenesis in isolated fat cells was performed as described in detail previously^67^. In brief, isolated fat cells were incubated for 2 hrs at 37 °C in a buffer containing unlabelled glucose and ^3^H-glucose without or with increasing concentrations of insulin. After incubation, lipids were extracted and radioactive glucose incorporation into total lipid was used as an index of lipogenesis. The ability of insulin to stimulate lipogenesis at maximum effective concentration was expressed as nmol glucose/10^7^ fat cells*2 hrs.

Adiponectin secretion from intact adipose tissue was determined in conditioned media (100 mg tissue/1 ml of medium) as described before^68^ except that an ELISA assay from Mercodia (Uppsala, Sweden) was used for the analyses^69^. Results were expressed as ng/10^7^ fat cells*2 hrs.

### Statistics

Values are presented as means ± S.E.M. Statistical evaluation was performed with the Mann–Whitney test using the GraphPad Prism software, version 5.0 (GraphPad Software, San Diego, CA, USA) or multiple regression using JMP (12.1 SAS Institute Inc., Cary, NC, USA). Non-normally distributed parameters were log_10_ transformed as indicated. Asterisks or pound signs denote statistically significant differences between the indicated groups of data (n.s. no significant; * or ^#^P < 0.05; ** or ^##^P < 0.01; *** or ^###^P < 0.001).

## Additional Information

**How to cite this article**: Peng, X. *et al*. Thioredoxin reductase 1 suppresses adipocyte differentiation and insulin responsiveness. *Sci. Rep.* **6**, 28080; doi: 10.1038/srep28080 (2016).

## Supplementary Material

Supplementary Information

## Figures and Tables

**Table 1:** Relationship between *TXNRD1* expression and insulin resistance *in vivo* and *in vitro*.

Parameter	*TXNRD1*		BMI	
	Std Beta Coefficient	P-value^a^	Std Beta Coefficient	P-value^a^
**KITT** (% fall in P-glucose/min at 4–16 min)	−0.44	0.0079 (**)	−0.36	0.030 (*)
**P-TG** (mM)^b^	0.18	0.22 (*n.s.*)	0.34	0.025 (*)
**Fat cell lipogenesis** (max insulin stimulated/10^7^ cells × 2 hrs)^b^	−0.38	0.0083 (**)	−0.31	0.032 (*)
**Adiponectin secretion** (ng/10^7^ cells × 2 hrs)^b^	0.11	0.50 (*n.s.*)	0.37	0.027 (*)

*TXNRD1* expression in subcutaneous adipose tissue from 56 individuals was determined by gene micro array and analyzed for possible correlation with clinical parameters. Multiple regression analysis was performed with *TXNRD1* and BMI set as Independent regressors with the listed parameters as dependent regressors. Standardized beta coefficients and p-values for the two independent regressors are displayed.

^a^Significance in p-values are indicated (**p-value < 0.01; *p-value < 0.05; *n.s*. = no significance, i.e. p-value > 0.05).

^b^For plasma triglycerides, fat cell lipogenesis and adiponectin secretion *in vitro*, values were log_10_ transformed to obtain normal distribution.

## References

[b1] Spalding K. L. . Dynamics of fat cell turnover in humans. Nature 453, 783–787 (2008). 18454136 10.1038/nature06902

[b2] Umek R. M., Friedman A. D. & McKnight S. L. CCAAT-enhancer binding protein: a component of a differentiation switch. Science 251, 288–292 (1991). 1987644 10.1126/science.1987644

[b3] Rosen E. D. & MacDougald O. A. Adipocyte differentiation from the inside out. Nat Rev Mol Cell Biol 7, 885–896 (2006). 17139329 10.1038/nrm2066

[b4] Rosen E. D., Walkey C. J., Puigserver P. & Spiegelman B. M. Transcriptional regulation of adipogenesis. Genes Dev 14, 1293–1307 (2000). 10837022

[b5] Tseng Y. H., Kriauciunas K. M., Kokkotou E. & Kahn C. R. Differential roles of insulin receptor substrates in brown adipocyte differentiation. Molecular and cellular biology 24, 1918–1929 (2004). 14966273 10.1128/MCB.24.5.1918-1929.2004 PMC350563

[b6] Laustsen P. G. . Lipoatrophic diabetes in Irs1(−/−)/Irs3(−/−) double knockout mice. Genes Dev 16, 3213–3222 (2002). 12502742 10.1101/gad.1034802 PMC187498

[b7] Garofalo R. S. . Severe diabetes, age-dependent loss of adipose tissue, and mild growth deficiency in mice lacking Akt2/PKB beta. J Clin Invest 112, 197–208 (2003). 12843127 10.1172/JCI16885 PMC164287

[b8] Arnér E. S. Focus on mammalian thioredoxin reductases–important selenoproteins with versatile functions. Biochim Biophys Acta 1790, 495–526 (2009). 19364476 10.1016/j.bbagen.2009.01.014

[b9] Bondareva A. A. . Effects of thioredoxin reductase-1 deletion on embryogenesis and transcriptome. Free radical biology & medicine 43, 911–923 (2007). 17697936 10.1016/j.freeradbiomed.2007.05.026 PMC2099259

[b10] Jakupoglu C. . Cytoplasmic thioredoxin reductase is essential for embryogenesis but dispensable for cardiac development. Mol Cell Biol 25, 1980–1988 (2005). 15713651 10.1128/MCB.25.5.1980-1988.2005 PMC549365

[b11] Iverson S. V. . A Txnrd1-dependent metabolic switch alters hepatic lipogenesis, glycogen storage, and detoxification. Free radical biology & medicine 63, 369–380 (2013). 23743293 10.1016/j.freeradbiomed.2013.05.028 PMC3827783

[b12] Carlson B. A. . Thioredoxin reductase 1 protects against chemically induced hepatocarcinogenesis via control of cellular redox homeostasis. Carcinogenesis 33, 1806–1813 (2012). 22791808 10.1093/carcin/bgs230 PMC3514905

[b13] Saeed H., Taipaleenmaki H., Aldahmash A. M., Abdallah B. M. & Kassem M. Mouse embryonic fibroblasts (MEF) exhibit a similar but not identical phenotype to bone marrow stromal stem cells (BMSC). Stem Cell Rev 8, 318–328 (2012). 21927803 10.1007/s12015-011-9315-x

[b14] Mandal P. K. . Loss of thioredoxin reductase 1 renders tumors highly susceptible to pharmacologic glutathione deprivation. Cancer Res 70, 9505–9514 (2010). 21045148 10.1158/0008-5472.CAN-10-1509

[b15] Peng X. . Sec-containing TrxR1 is essential for self-sufficiency of cells by control of glucose-derived H2O2. Cell Death Dis 5, e1235 (2014). 24853413 10.1038/cddis.2014.209 PMC4047868

[b16] Ross S. E. . Inhibition of adipogenesis by Wnt signaling. Science 289, 950–953 (2000). 10937998 10.1126/science.289.5481.950

[b17] Tang Q. Q., Otto T. C. & Lane M. D. Mitotic clonal expansion: a synchronous process required for adipogenesis. P Natl Acad Sci USA 100, 44–49 (2003). 10.1073/pnas.0137044100 PMC140878 12502791

[b18] Tang Q. Q., Otto T. C. & Lane M. D. CCAAT/enhancer-binding protein beta is required for mitotic clonal expansion during adipogenesis. P Natl Acad Sci USA 100, 850–855 (2003). 10.1073/pnas.0337434100 PMC298690 12525691

[b19] Lechner S., Mitterberger M. C., Mattesich M. & Zwerschke W. Role of C/EBPbeta-LAP and C/EBPbeta-LIP in early adipogenic differentiation of human white adipose-derived progenitors and at later stages in immature adipocytes. Differentiation 85, 20–31 (2013). 23314288 10.1016/j.diff.2012.11.001

[b20] Taniguchi C. M., Emanuelli B. & Kahn C. R. Critical nodes in signalling pathways: insights into insulin action. Nat Rev Mol Cell Biol 7, 85–96 (2006). 16493415 10.1038/nrm1837

[b21] Du K. & Montminy M. CREB is a regulatory target for the protein kinase Akt/PKB. The Journal of biological chemistry 273, 32377–32379 (1998). 9829964 10.1074/jbc.273.49.32377

[b22] Reusch J. E., Colton L. A. & Klemm D. J. CREB activation induces adipogenesis in 3T3-L1 cells. Molecular and cellular biology 20, 1008–1020 (2000). 10629058 10.1128/mcb.20.3.1008-1020.2000 PMC85218

[b23] Shaywitz A. J. & Greenberg M. E. CREB: a stimulus-induced transcription factor activated by a diverse array of extracellular signals. Annual review of biochemistry 68, 821–861 (1999). 10.1146/annurev.biochem.68.1.821 10872467

[b24] Kim J. E. & Chen J. regulation of peroxisome proliferator-activated receptor-gamma activity by mammalian target of rapamycin and amino acids in adipogenesis. Diabetes 53, 2748–2756 (2004). 15504954 10.2337/diabetes.53.11.2748

[b25] Porstmann T. . SREBP activity is regulated by mTORC1 and contributes to Akt-dependent cell growth. Cell Metab 8, 224–236 (2008). 18762023 10.1016/j.cmet.2008.07.007 PMC2593919

[b26] Dagnell M. . Selective activation of oxidized PTP1B by the thioredoxin system modulates PDGF-beta receptor tyrosine kinase signaling. P Natl Acad Sci USA 110, 13398–13403 (2013). 10.1073/pnas.1302891110 PMC3746926 23901112

[b27] Elchebly M. . Increased insulin sensitivity and obesity resistance in mice lacking the protein tyrosine phosphatase-1B gene. Science 283, 1544–1548 (1999). 10066179 10.1126/science.283.5407.1544

[b28] Tamguney T. & Stokoe D. New insights into PTEN. Journal of cell science 120, 4071–4079 (2007). 18032782 10.1242/jcs.015230

[b29] Stambolic V. . Regulation of PTEN transcription by p53. Mol Cell 8, 317–325 (2001). 11545734 10.1016/s1097-2765(01)00323-9

[b30] Tang Y. & Eng C. PTEN autoregulates its expression by stabilization of p53 in a phosphatase-independent manner. Cancer research 66, 736–742 (2006). 16424003 10.1158/0008-5472.CAN-05-1557

[b31] Moos P. J., Edes K., Cassidy P., Massuda E. & Fitzpatrick F. A. Electrophilic prostaglandins and lipid aldehydes repress redox-sensitive transcription factors p53 and hypoxia-inducible factor by impairing the selenoprotein thioredoxin reductase. The Journal of biological chemistry 278, 745–750 (2003). 12424231 10.1074/jbc.M211134200

[b32] Meuillet E. J., Mahadevan D., Berggren M., Coon A. & Powis G. Thioredoxin-1 binds to the C2 domain of PTEN inhibiting PTEN’s lipid phosphatase activity and membrane binding: a mechanism for the functional loss of PTEN’s tumor suppressor activity. Archives of biochemistry and biophysics 429, 123–133 (2004). 15313215 10.1016/j.abb.2004.04.020

[b33] Lee S. R. . Reversible inactivation of the tumor suppressor PTEN by H2O2. The Journal of biological chemistry 277, 20336–20342 (2002). 11916965 10.1074/jbc.M111899200

[b34] Schwertassek U. . Reactivation of oxidized PTP1B and PTEN by thioredoxin 1. Febs J 281, 3545–3558 (2014). 24976139 10.1111/febs.12898 PMC4162488

[b35] Kanda Y., Hinata T., Kang S. W. & Watanabe Y. Reactive oxygen species mediate adipocyte differentiation in mesenchymal stem cells. Life Sci 89, 250–258 (2011). 21722651 10.1016/j.lfs.2011.06.007

[b36] Samuni Y., Goldstein S., Dean O. M. & Berk M. The chemistry and biological activities of N-acetylcysteine. Biochimica et biophysica acta 1830, 4117–4129 (2013). 23618697 10.1016/j.bbagen.2013.04.016

[b37] Polyak K. . Cloning of p27Kip1, a cyclin-dependent kinase inhibitor and a potential mediator of extracellular antimitogenic signals. Cell 78, 59–66 (1994). 8033212 10.1016/0092-8674(94)90572-x

[b38] Fujita N., Sato S., Katayama K. & Tsuruo T. Akt-dependent phosphorylation of p27Kip1 promotes binding to 14-3-3 and cytoplasmic localization. The Journal of biological chemistry 277, 28706–28713 (2002). 12042314 10.1074/jbc.M203668200

[b39] Shin I. . PKB/Akt mediates cell-cycle progression by phosphorylation of p27(Kip1) at threonine 157 and modulation of its cellular localization. Nature medicine 8, 1145–1152 (2002). 10.1038/nm759 12244301

[b40] Schopfer F. J. . Nitrolinoleic acid: an endogenous peroxisome proliferator-activated receptor gamma ligand. P Natl Acad Sci USA 102, 2340–2345 (2005). 10.1073/pnas.0408384102 PMC548962 15701701

[b41] Freeman B. A. . Nitro-fatty acid formation and signaling. The Journal of biological chemistry 283, 15515–15519 (2008). 18285326 10.1074/jbc.R800004200 PMC2414282

[b42] Schopfer F. J. . Covalent peroxisome proliferator-activated receptor gamma adduction by nitro-fatty acids: selective ligand activity and anti-diabetic signaling actions. The Journal of biological chemistry 285, 12321–12333 (2010). 20097754 10.1074/jbc.M109.091512 PMC2852971

[b43] Arner E. . Adipose tissue microRNAs as regulators of CCL2 production in human obesity. Diabetes 61, 1986–1993 (2012). 22688341 10.2337/db11-1508 PMC3402332

[b44] Acin-Perez R. . ROS-triggered phosphorylation of complex II by Fgr kinase regulates cellular adaptation to fuel use. Cell Metab 19, 1020–1033 (2014). 24856931 10.1016/j.cmet.2014.04.015 PMC4274740

[b45] Zhang H. H. . Insulin stimulates adipogenesis through the Akt-TSC2-mTORC1 pathway. PLoS One 4, e6189 (2009). 19593385 10.1371/journal.pone.0006189 PMC2703782

[b46] Molchadsky A. . p53 plays a role in mesenchymal differentiation programs, in a cell fate dependent manner. PLoS One 3, e3707 (2008). 19002260 10.1371/journal.pone.0003707 PMC2577894

[b47] Du Y., Zhang H., Lu J. & Holmgren A. Glutathione and glutaredoxin act as a backup of human thioredoxin reductase 1 to reduce thioredoxin 1 preventing cell death by aurothioglucose. The Journal of biological chemistry 287, 38210–38219 (2012). 22977247 10.1074/jbc.M112.392225 PMC3488090

[b48] Mandal P. K. . System x(c)- and thioredoxin reductase 1 cooperatively rescue glutathione deficiency. The Journal of biological chemistry 285, 22244–22253 (2010). 20463017 10.1074/jbc.M110.121327 PMC2903358

[b49] Zhang J. . Selective disruption of PPARgamma 2 impairs the development of adipose tissue and insulin sensitivity. P Natl Acad Sci USA 101, 10703–10708 (2004). 10.1073/pnas.0403652101 PMC489998 15249658

[b50] Enerback S. The origins of brown adipose tissue. N Engl J Med 360, 2021–2023 (2009). 19420373 10.1056/NEJMcibr0809610

[b51] Tormos K. V. . Mitochondrial complex III ROS regulate adipocyte differentiation. Cell Metab 14, 537–544 (2011). 21982713 10.1016/j.cmet.2011.08.007 PMC3190168

[b52] Lee H., Lee Y. J., Choi H., Ko E. H. & Kim J. W. Reactive oxygen species facilitate adipocyte differentiation by accelerating mitotic clonal expansion. The Journal of biological chemistry 284, 10601–10609 (2009). 19237544 10.1074/jbc.M808742200 PMC2667747

[b53] Menon S. G. . Redox regulation of the G1 to S phase transition in the mouse embryo fibroblast cell cycle. Cancer research 63, 2109–2117 (2003). 12727827

[b54] Ibanez I. L. . H2O2 scavenging inhibits G1/S transition by increasing nuclear levels of p27KIP1. Cancer letters 305, 58–68 (2011). 21411221 10.1016/j.canlet.2011.02.026

[b55] Burhans W. C. & Heintz N. H. The cell cycle is a redox cycle: linking phase-specific targets to cell fate. Free radical biology & medicine 47, 1282–1293 (2009). 19486941 10.1016/j.freeradbiomed.2009.05.026

[b56] Rassidakis G. Z. . Inhibition of Akt increases p27Kip1 levels and induces cell cycle arrest in anaplastic large cell lymphoma. Blood 105, 827–829 (2005). 15374880 10.1182/blood-2004-06-2125 PMC1382060

[b57] Ushio-Fukai M. . Reactive oxygen species mediate the activation of Akt/protein kinase B by angiotensin II in vascular smooth muscle cells. The Journal of biological chemistry 274, 22699–22704 (1999). 10428852 10.1074/jbc.274.32.22699

[b58] Hou J. . Reactive oxygen species-mediated activation of the Src-epidermal growth factor receptor-Akt signaling cascade prevents bortezomib-induced apoptosis in hepatocellular carcinoma cells. Molecular medicine reports 11, 712–718 (2015). 25338626 10.3892/mmr.2014.2736

[b59] Berger J. & Moller D. E. The mechanisms of action of PPARs. Annu Rev Med 53, 409–435 (2002). 11818483 10.1146/annurev.med.53.082901.104018

[b60] Ramaswamy S. . Regulation of G1 progression by the PTEN tumor suppressor protein is linked to inhibition of the phosphatidylinositol 3-kinase/Akt pathway. P Natl Acad Sci USA 96, 2110–2115 (1999). 10.1073/pnas.96.5.2110 PMC26745 10051603

[b61] Van Harmelen V., Skurk T. & Hauner H. Primary culture and differentiation of human adipocyte precursor cells. Methods Mol Med 107, 125–135 (2005). 15492368 10.1385/1-59259-861-7:125

[b62] Lorente-Cebrian S. . MicroRNAs regulate human adipocyte lipolysis: effects of miR-145 are linked to TNF-alpha. PLoS One 9, e86800 (2014). 24475180 10.1371/journal.pone.0086800 PMC3901697

[b63] Gimenez-Cassina A. . Regulation of hepatic energy metabolism and gluconeogenesis by BAD. Cell Metab 19, 272–284 (2014). 24506868 10.1016/j.cmet.2013.12.001 PMC3971904

[b64] Simarro M. . Fast kinase domain-containing protein 3 is a mitochondrial protein essential for cellular respiration. Biochem Biophys Res Commun 401, 440–446 (2010). 20869947 10.1016/j.bbrc.2010.09.075 PMC2963690

[b65] Riddles P. W., Blakeley R. L. & Zerner B. Reassessment of Ellman’s reagent. Methods in enzymology 91, 49–60 (1983). 6855597 10.1016/s0076-6879(83)91010-8

[b66] Lorente-Cebrian S. . Allograft inflammatory factor 1 (AIF-1) is a new human adipokine involved in adipose inflammation in obese women. BMC Endocr Disord 13, 54 (2013). 24267103 10.1186/1472-6823-13-54 PMC4175115

[b67] Kaaman M. . Strong association between mitochondrial DNA copy number and lipogenesis in human white adipose tissue. Diabetologia (2007). 10.1007/s00125-007-0818-6 17879081

[b68] Hoffstedt J., Arvidsson E., Sjolin E., Wahlen K. & Arner P. Adipose tissue adiponectin production and adiponectin serum concentration in human obesity and insulin resistance. J Clin Endocrinol Metab 89, 1391–1396 (2004). 15001639 10.1210/jc.2003-031458

[b69] Belarbi Y. . MicroRNA-193b Controls Adiponectin Production in Human White Adipose Tissue. J Clin Endocrinol Metab 100, E1084–1088 (2015). 26020766 10.1210/jc.2015-1530

[b70] Zhang X. . Disruption of the mitochondrial thioredoxin system as a cell death mechanism of cationic triphenylmethanes. Free radical biology & medicine 50, 811–820 (2011). 21215310 10.1016/j.freeradbiomed.2010.12.036 PMC3047390

