# Supplementary material for: Thioredoxin reductase 1 suppresses adipocyte differentiation and insulin responsiveness
Source: Sci Rep. 2016 Jun 27;6:28080. doi: 10.1038/srep28080 (PMC4921861; doi:10.1038/srep28080)
Supplement: Supplementary Information [file srep28080-s1.pdf]

Thioredoxin reductase 1 suppresses adipocyte differentiation and insulin responsiveness

Xiaoxiao Peng[1§], Alfredo Giménez-Cassina[1], Paul Petrus[2], Marcus Conrad[3], Mikael Rydén[2] and Elias S.J. Arnér*[,1]

[1] Division of Biochemistry, Department of Medical Biochemistry and Biophysics, Karolinska Institutet, SE-171 77 Stockholm, Sweden;
[2] Clinical Research Center, and the Department of Medicine, Huddinge University Hospital, Karolinska Institutet, SE-141 86 Stockholm, Sweden;
[3] Helmholtz Zentrum München, Institute of Developmental Genetics, Ingolstädter Landstrasse 1, 85764 Neuherberg, Germany
[§] Present address: CVMD Translational Medicine Unit, Early Clinical Development, AstraZeneca, Mölndal, Sweden.

Supplementary material: Original non-cropped images of western blots

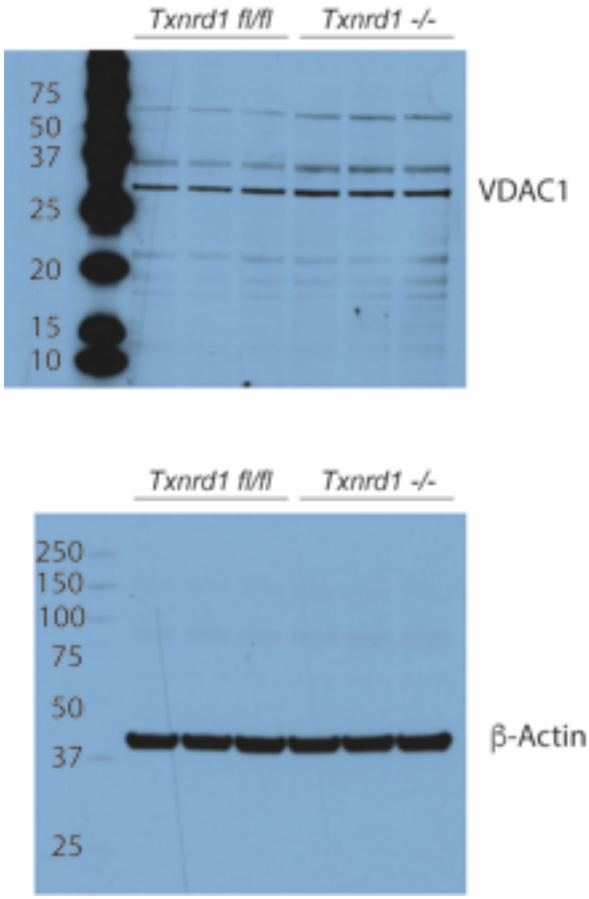

Non-cropped blots for the experiments shown in Fig. 1D of the main article.

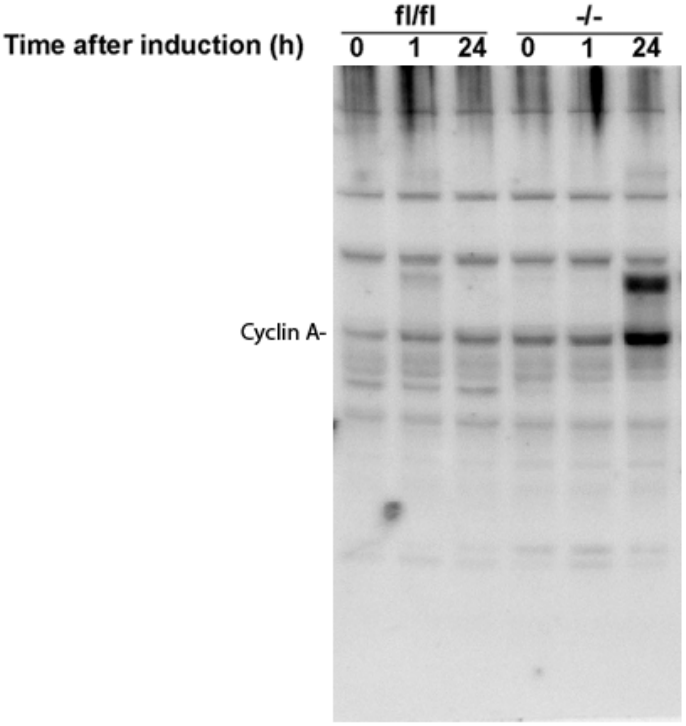

Non-cropped blot for the experiment shown in Fig. 3B of the main article.

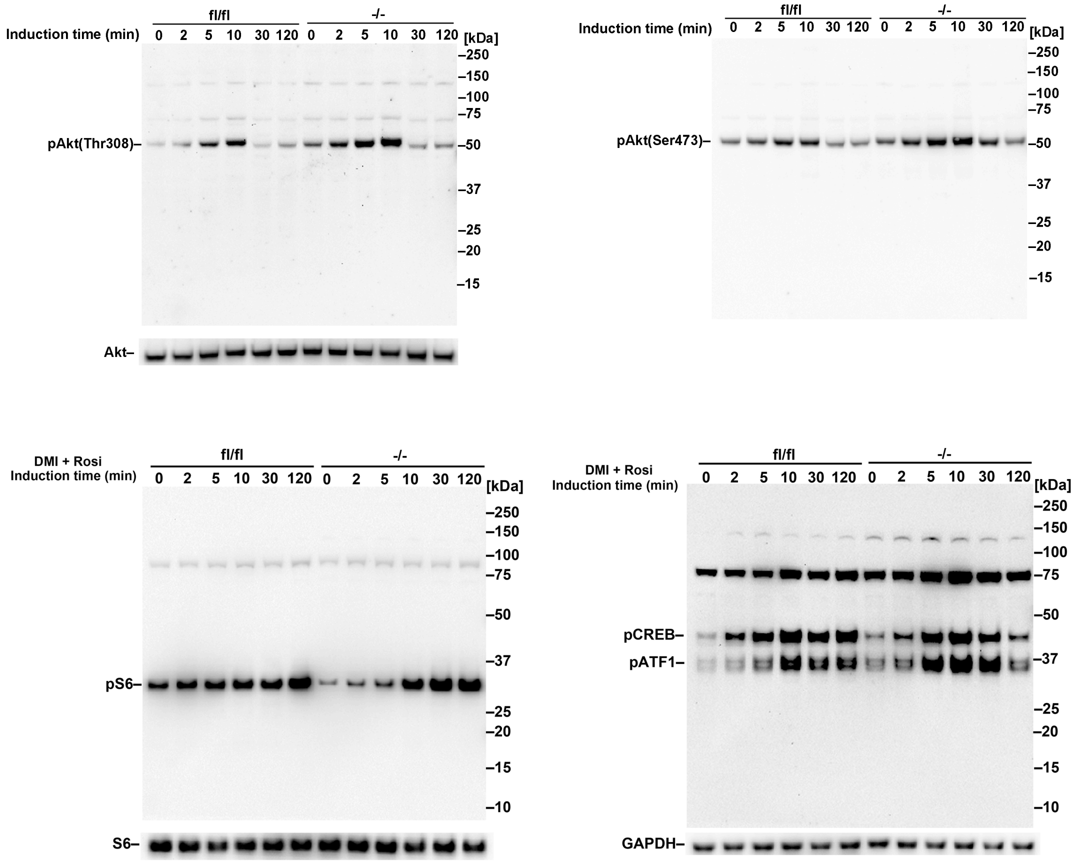

Non-cropped blots for the experiments shown in Fig. 3C of the main article.

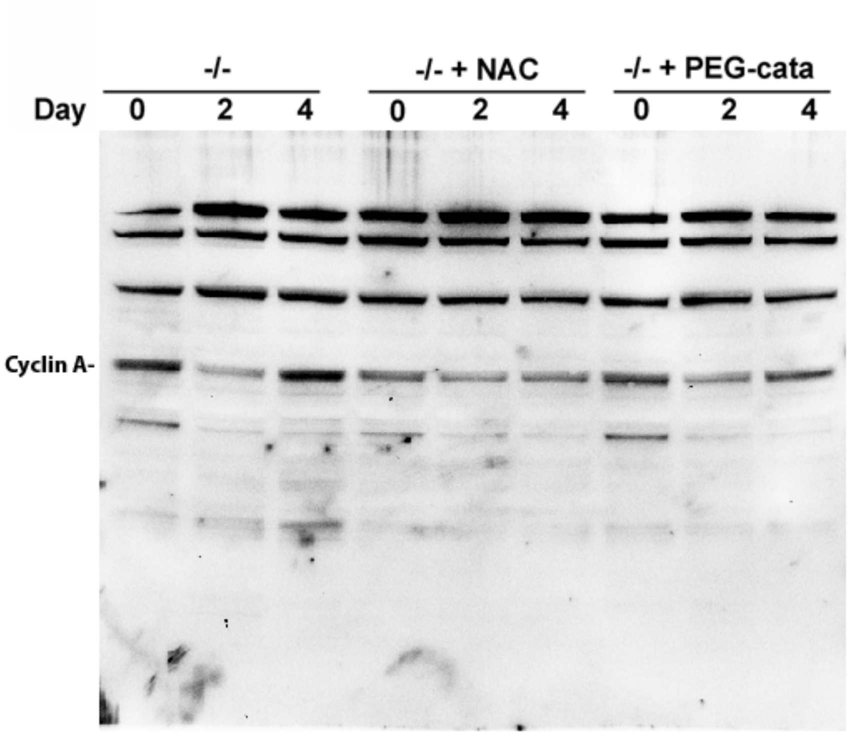

Non-cropped blot for the experiment shown in Fig. 5F of the main article.

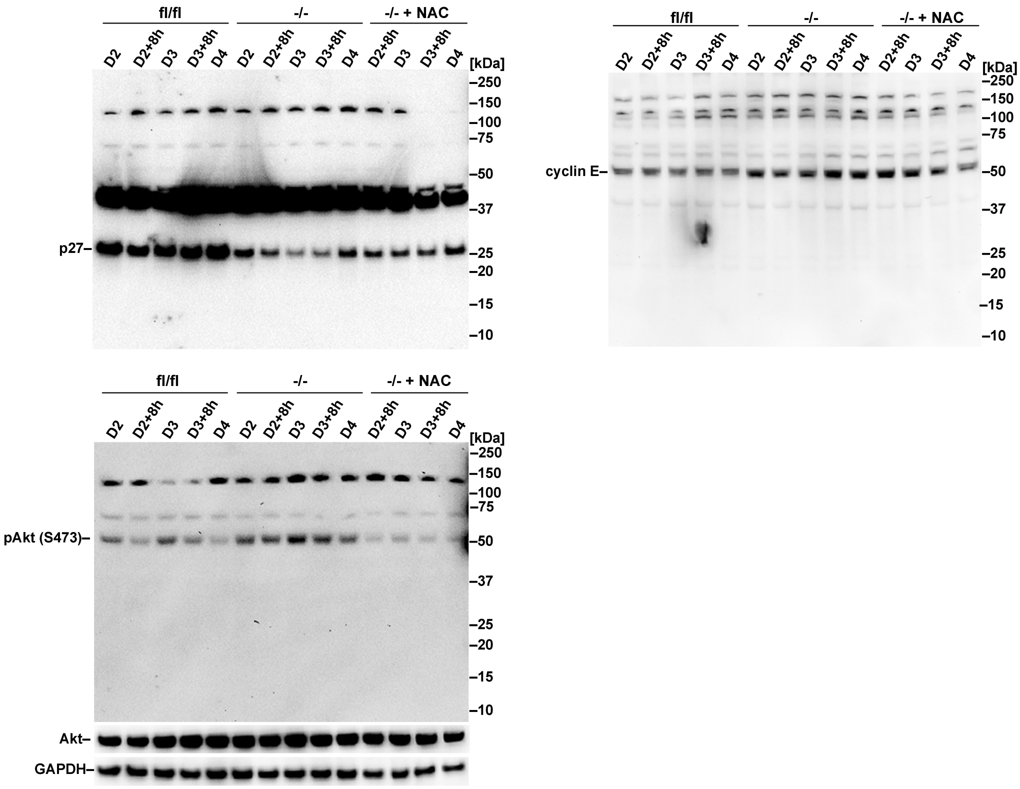

Non-cropped blots for the experiments shown in Fig. 5H of the main article.
